# Lateral attachment of kinetochores to microtubules is enriched in prometaphase rosette and facilitates chromosome alignment and bi-orientation establishment

**DOI:** 10.1038/s41598-018-22164-5

**Published:** 2018-03-01

**Authors:** Go Itoh, Masanori Ikeda, Kenji Iemura, Mohammed Abdullahel Amin, Sei Kuriyama, Masamitsu Tanaka, Natsuki Mizuno, Hiroko Osakada, Tokuko Haraguchi, Kozo Tanaka

**Affiliations:** 1 Department of Molecular Oncology, Institute of Development, Aging and Cancer, Tohoku University, Sendai, 980-8575 Japan; 2 Department of Molecular Medicine and Biochemistry, Akita University Graduate School of Medicine, Akita, 010-8543 Japan; 3 Advanced ICT Research Institute, National Institute of Information and Communications Technology (NICT), Kobe, 651-2492 Japan; 4 Graduate School of Frontier Biosciences, Osaka University, Suita, 565-0871 Japan; 5 Graduate School of Science, Osaka University, Toyonaka, 560-0043 Japan; 6 Present Address: Department of Cell and Molecular Biology, Feinberg School of Medicine, Northwestern University, Chicago, IL 60611 USA

## Abstract

Faithful chromosome segregation is ensured by the establishment of bi-orientation; the attachment of sister kinetochores to the end of microtubules extending from opposite spindle poles. In addition, kinetochores can also attach to lateral surfaces of microtubules; called lateral attachment, which plays a role in chromosome capture and transport. However, molecular basis and biological significance of lateral attachment are not fully understood. We have addressed these questions by focusing on the prometaphase rosette, a typical chromosome configuration in early prometaphase. We found that kinetochores form uniform lateral attachments in the prometaphase rosette. Many transient kinetochore components are maximally enriched, in an Aurora B activity-dependent manner, when the prometaphase rosette is formed. We revealed that rosette formation is driven by rapid poleward motion of dynein, but can occur even in its absence, through slow kinetochore movements caused by microtubule depolymerization that is supposedly dependent on kinetochore tethering at microtubule ends by CENP-E. We also found that chromosome connection to microtubules is extensively lost when lateral attachment is perturbed in cells defective in end-on attachment. Our findings demonstrate that lateral attachment is an important intermediate in bi-orientation establishment and chromosome alignment, playing a crucial role in incorporating chromosomes into the nascent spindle.

## Introduction

For faithful chromosome segregation in mitosis, kinetochores on all the sister chromatid pairs have to establish bipolar attachment, or bi-orientation, which is the attachment of sister kinetochores to microtubules emanating from opposite spindle poles^1^. On bi-oriented kinetochores, bundles of 20–30 microtubules, known as k-fibers, attach with their ends terminating at the kinetochore, in a manner called end-on attachment. This enables chromosome motion by the elongation and shrinkage of the k-fibers. In contrast, kinetochores can also attach to the sides of microtubules, referred to as lateral attachment, and move along microtubules mediated by the activities of motor proteins. The mechanism is conserved from yeast to humans^2^. Kinetochores are efficiently captured by the lateral surface of microtubules and transported towards spindle poles^2^ driven, in higher eukaryotes, by dynein^3,4^. Recent studies revealed that lateral attachment in higher eukaryotes also plays a role in the accumulation of chromosomes to the spindle equator before they align on the so-called metaphase plate^5–7^. We have recently reported that two motor proteins, Kid and CENP-E, play differential roles in this process^8^. It has been suggested that bi-orientation is efficiently established for the chromosomes transported to the spindle equator through lateral attachment^7,9^. These findings imply that lateral attachment is not just a transient, unstable initial attachment but an important intermediate for development of bi-orientation. However, end-on attachments frequently seem to be formed directly and not through lateral attachment^10,11^. Thus, the molecular mechanisms and biological significance of lateral attachment are not fully understood.

It has been known that, during prometaphase, chromosomes often show a characteristic convex arrangement, originally called the ‘prometaphase configuration’^12^ or ‘prometaphase rosette’^13,14^. It was once proposed that chromosomes were distributed non-randomly in the prometaphase rosette^13^, but this idea has been challenged in later studies^14^. However, it has not been directly addressed how the prometaphase rosette is formed and how kinetochores attach to microtubules within it. Focusing on the prometaphase rosette, we addressed the molecular basis and biological significance of lateral attachment. We found that the prometaphase rosette is composed of chromosomes laterally attaching to the nascent spindle. The majority of the transient kinetochore components maximally localize to kinetochores when the prometaphase rosette is formed, and such localization is mainly dependent on Aurora B activity. Formation of the prometaphase rosette is driven by rapid poleward motion of dynein. However, in the absence of dynein, CENP-E-dependent kinetochore tethering to microtubule ends allows a slow formation of the prometaphase rosette. Furthermore, we found that when lateral attachments are suppressed together with end-on attachments, kinetochore attachments to microtubules are extensively lost. Our data suggest that lateral attachment plays a pivotal role in bi-orientation establishment through the efficient incorporation of chromosomes to the spindle.

## Results

### Kinetochores are laterally attached to microtubules in the prometaphase rosette

**Figure 1:**
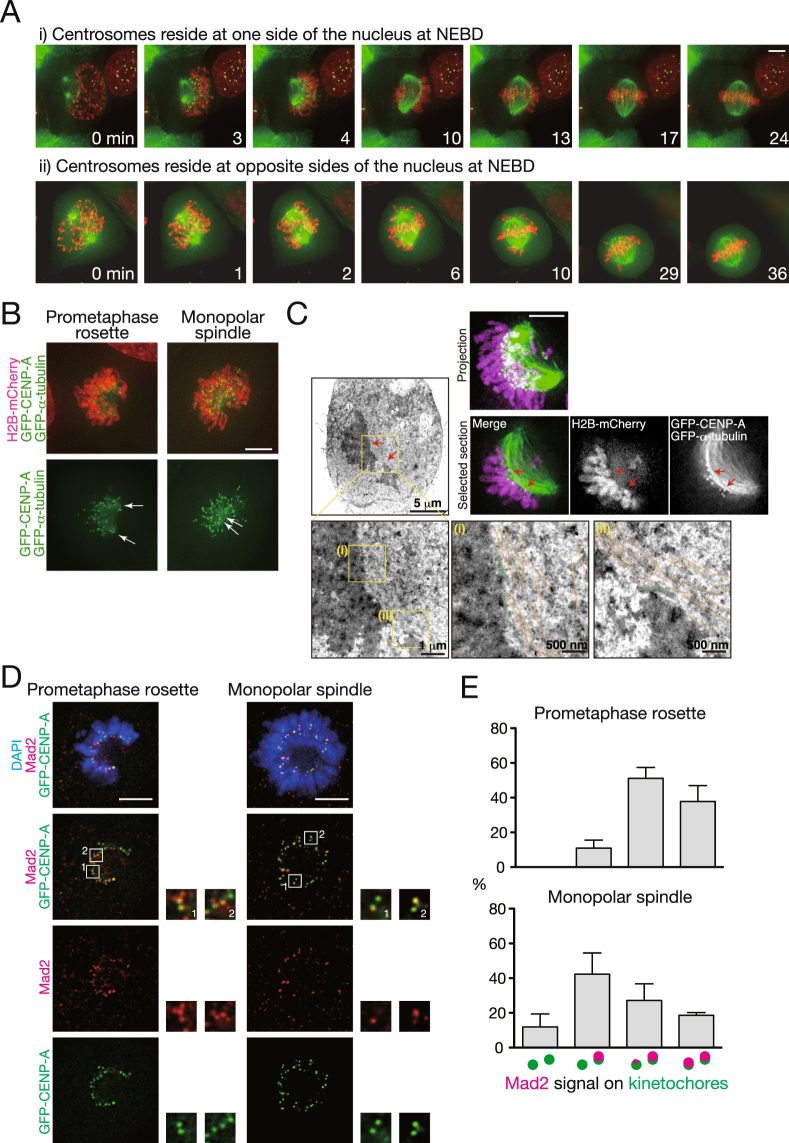
Kinetochores laterally associate with microtubules in the prometaphase rosette. (**A**) Process of chromosome alignment depending on the position of the centrosomes at nuclear envelope breakdown (NEBD). HeLa cells expressing EGFP–α-tubulin (green), EGFP–CENP-A (green), and H2B–mCherry (red) were imaged at 1 min intervals, starting from NEBD. A cell in which centrosomes reside at one side (i) or opposite sides (ii) of the nucleus at NEBD is shown. Scale bar: 5 μm. (**B**) Comparison between the prometaphase rosette (left) and monopolar spindle (right). HeLa cells expressing EGFP–α-tubulin (green), EGFP–CENP-A (green), and H2B–mCherry (red) were observed. Monopolar spindles were formed by inhibiting Eg5 with monastrol. Positions of centrosomes are shown by arrows. Scale bar: 5 μm. (**C**) Lateral attachment of kinetochores to microtubules in the prometaphase rosette. HeLa cells expressing EGFP–α-tubulin (green), EGFP–CENP-A (green), and H2B–mCherry (magenta) were observed by both fluorescence microscopy and transmission electron microscopy. Boxed areas in the electron microscopy image are magnified in the right panels. Size of scale bar is shown in each panel. Positions of kinetochores and microtubules are highlighted by green and orange lines, respectively. Fluorescence microscopy images of the same cell are shown in the upper right panels. Corresponding areas magnified in the electron microscopy images are indicated by arrows. Scale bar: 5 μm. (**D**) Mad2 localization on kinetochores in the prometaphase rosette (left) and monopolar spindle (right). HeLa cells expressing EGFP–CENP-A were immunostained with an antibody against Mad2 (red). DNA was stained with DAPI (blue). Monopolar spindles were formed by inhibiting Eg5 with monastrol. Scale bars: 5 μm. (**E**) Pattern of kinetochore localization of Mad2 in the prometaphase rosette (upper) and monopolar spindle (lower). Percentage of sister kinetochore pairs showing each pattern of Mad2 signal as schematized is shown. Representative data from three independent experiments are presented. At least 101 kinetochore pairs from 3 cells were observed for each condition. Error bars represent S.D.

First we addressed how the prometaphase rosette is formed. We observed HeLa cells expressing EGFP–α-tubulin, EGFP–CENP-A, and H2B–mCherry to visualize microtubules, kinetochores, and chromosomes, respectively, by live cell imaging. We found that the prometaphase rosette is formed when centrosomes reside at the same side of the nucleus at nuclear envelope breakdown (NEBD) (Fig. 1A–i, Supplementary Movie 1). After NEBD, chromosomes quickly assemble around the nascent spindle (Fig. 1A–i, 3 min), and cover the spindle surface (Fig. 1A–i, 4 min). The chromosomes then move to the spindle equator (Fig. 1A–i, 10–17 min), forming the metaphase plate (Fig. 1A–i, 24 min). Formation of the prometaphase rosette around the nascent spindle and subsequent formation of the metaphase plate is also shown in Supplementary Fig. S1A. In contrast, when centrosomes reside at opposite sides of the nucleus at NEBD, chromosomes locating between centrosomes (now referred to as spindle poles) directly move to the equator of the nascent spindle, whereas chromosomes outside of the spindle assemble to its surface (Fig. 1A–ii, 1–2 min, Supplementary Movie 2); thus the typical prometaphase rosette is not formed in this case. Chromosomes on the surface of the spindle move to the spindle equator (Fig. 1A–ii, 6–29 min) before forming the metaphase plate (Fig. 1A–ii, 36 min), similarly to the situation in which both centrosomes are at one side at NEBD. These two types of chromosome alignment appeared at a similar frequency (86/190 (45.5%) vs 104/190 (54.5%)), in agreement with a previous report^15^. In the report, the two alignment patterns correspond to a prophase pathway and a prometaphase pathway, respectively, based on the timing of centrosome separation^16^. Actually, even when centrosomes look un-separated in projected images they are, in fact, well separated on different focal planes (Supplementary Fig. S1B), as pointed out previously^7^. These results suggest that the prometaphase rosette is formed when the axis connecting the centrosomes is outside of the nucleus at NEBD and thus chromosomes are not directly incorporated into the nascent spindle after NEBD.

Chromosome configuration in the prometaphase rosette looks similar to a monopolar spindle, in which centrosomes are not separated. This is typically seen in cells treated with monastrol, an inhibitor against Eg5, a kinesin-5 motor protein required for the bipolarity of the spindle (Fig. 1B). But, as mentioned above, centrosomes are separated in the prometaphase rosette (Fig. 1B, Supplementary Fig. S1B), whereas in a typical monopolar spindle chromosomes form a circle with coalesced centrosomes at the center (Fig. 1B). In contrast, chromosomes in the prometaphase rosette do not form a perfect circle, but show a crescent shape (Fig. 1B). Centrosomes are at the gap in the chromosome arc, and microtubules elongating from the centrosomes bend and interdigitate, forming the nascent spindle to which chromosomes attach at the surface.

In a monopolar spindle, kinetochores usually form end-on attachments to microtubules (Fig. 1B)^17^. We examined how kinetochores attach to microtubules in the prometaphase rosette by correlative light and electron microscopy (CLEM). Cells showing the prometaphase rosette by fluorescence microscopy were fixed, and kinetochore–microtubule attachments in these cells were examined by electron microscopy. As shown in Fig. 1C, kinetochores attach to the side, not the end, of microtubules, showing that kinetochores form lateral attachment to microtubules in the prometaphase rosette, in contrast to kinetochores in monopolar spindle. Next we examined the localization of Mad2 on kinetochores in the prometaphase rosette and monopolar spindle. Mad2 is a component of the spindle assembly checkpoint (SAC), and localizes to kinetochores before stable end-on attachment is formed. In monopolar spindles formed by monastrol treatment, sister kinetochores are often arranged along the line connecting them to the coalesced spindle poles. In most cases, Mad2 was seen on the sister kinetochore most distant from the spindle pole while no, or weak, signal was seen on its sister kinetochore (Fig. 1D,E). This indicates that the sister kinetochore facing the spindle pole forms end-on attachment whereas the other does not, as shown previously^17^. In contrast, in the prometaphase rosette sister kinetochores were arranged perpendicularly to the line connecting them to spindle poles, and Mad2 staining was seen in both sister kinetochores in most cases, suggesting that end-on attachment is not formed (Fig. 1D,E).

### The process of chromosome alignment correlates with the transition of kinetochore–microtubule interaction

**Figure 2:**
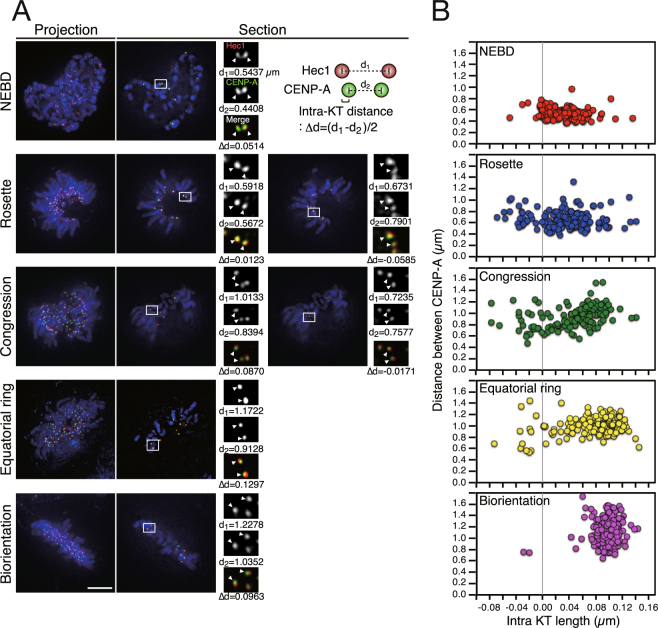
Inter- and intra-kinetochore distance in each phase of chromosome alignment. (**A**) Phases of chromosome alignment when centrosomes reside at one side of the nucleus at NEBD. HeLa cells expressing EGFP–CENP-A (green) were immunostained with an antibody against Hec1 (red). DNA was stained with DAPI (blue). Representative images of a cell for each phase are shown both as a projected image and single section, and a magnified view of a kinetochore pair boxed in the panel is shown in insets. As schematically shown, for each kinetochore pair indicated by white arrowheads, distance between Hec1 signals (d1), distance between CENP-A signals (d2), and intra-kinetochore distance (Δd) are indicated. Scale bar: 5 μm. (**B**) Quantification of inter- and intra-kinetochore distance in each phase of chromosome alignment. Distance between CENP-A signals (d2) and intra-kinetochore distance of sister kinetochore pairs (Δd) are plotted. Representative data from three independent experiments are presented. At least 30 kinetochore pairs from 5 cells were observed for each phase.

It has been reported that lateral attachment is predominant during early prometaphase, and then laterally attached chromosomes move towards the spindle equator where lateral attachment is converted to stable end-on attachment^7^. Our observation suggests that formation of the prometaphase rosette is a step in chromosome alignment when lateral attachment is maximally enriched. To verify this possibility, we first categorized the process of chromosome alignment into five distinct phases: NEBD, prometaphase rosette, congression, equatorial ring, and bi-orientation (Fig. 2A). The equatorial ring was defined previously as the chromosome ring formed at the spindle equator in which chromosomes are laterally attached to microtubules (Fig. 2A)^7^. We categorized the phase between the prometaphase rosette and the equatorial ring as congression phase, in which chromosomes move along the spindle surface towards the spindle equator (Fig. 2A). When lateral attachment in the equatorial ring is converted to end-on attachment, during which bi-orientation is established, the chromosome ring is transformed into the metaphase plate, filling the space inside the ring with chromosomes (Fig. 2A)^7^.

To evaluate kinetochore–microtubule interaction in each of the step in the process of chromosome alignment, we measured both intra- and inter-kinetochore distances. Inter-kinetochore distance reflects the tension exerted between sister kinetochores; it becomes bigger when kinetochores form bi-orientation. Intra-kinetochore distance reflects the kinetochore deformation that is related to the end-on attachment to dynamic microtubules^18,19^. Inter-kinetochore distance was measured as the distance between centroids of CENP-A signals on sister kinetochores, while intra-kinetochore distance was calculated as the half of the difference between Hec1 pair distance and CENP-A pair distance (Fig. 2A). At NEBD, both inter- and intra-kinetochore distance were small, indicating that kinetochores are not attached to microtubules (Fig. 2B). In the prometaphase rosette, inter- and intra-kinetochore distances were almost the same as that at NEBD (Fig. 2B), suggesting that tension was not applied on laterally attached kinetochores in the prometaphase rosette. Interestingly, distance between CENP-A signals was bigger than that between Hec1 signals in a fraction of kinetochore pairs, supposedly reflecting the structural change upon lateral attachment (Fig. 2A,B). The structural change of kinetochores may be related to “kinetochore swivel”, which was recently reported^20^. During congression phase, both inter- and intra-kinetochore distances increased, consistent with a previous report that inter-kinetochore distance increased to some extent during lateral attachment (Fig. 2B)^7^. In the equatorial ring phase, inter- and intra-kinetochore distance increased further (Fig. 2B). Finally, at bi-orientation, inter-kinetochore distance was maximal and kinetochores with negative or small intra-kinetochore distance disappeared, suggesting that most of the kinetochores formed stable end-on attachments (Fig. 2B). These data suggest that each phase of the chromosome alignment reflects a transition in the mode of kinetochore–microtubule attachment, and kinetochores uniformly form lateral attachments in the prometaphase rosette.

### Dynamic localization of transient kinetochore components during chromosome alignment

**Figure 3:**
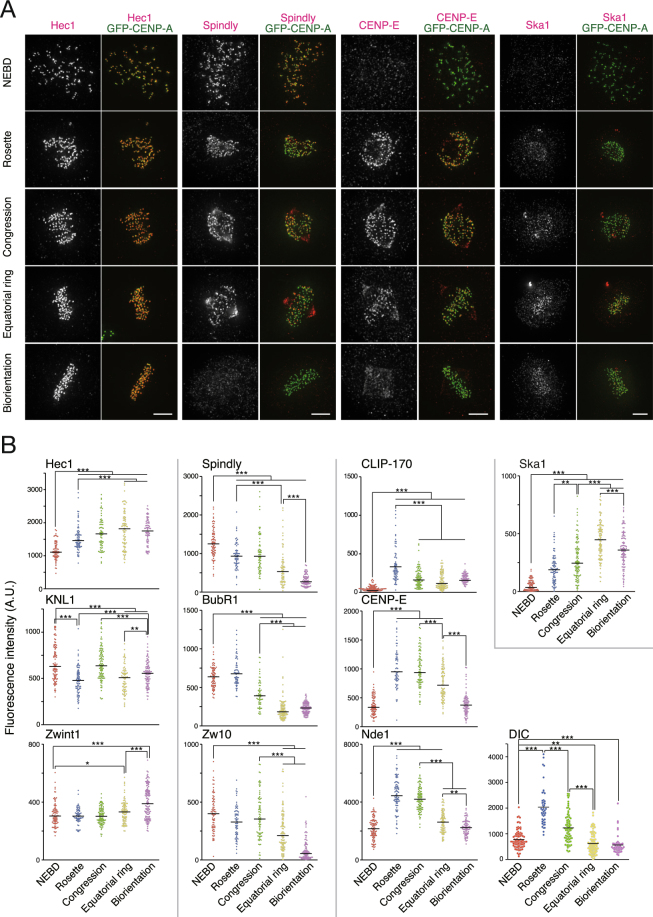
Localization profiles of kinetochore components in each phase of early mitosis. (**A**) Kinetochore localization of molecules showing different localization patterns during the process of chromosome alignment. HeLa cells expressing EGFP–CENP-A (green) were immunostained with an antibody against each kinetochore component (red), as indicated. Scale bar: 5 μm. (**B**) Quantification of the signal intensity of kinetochore components in the process of chromosome alignment. Molecules showing similar localization profiles are grouped, and separated by gray lines from other groups. At least 30 kinetochore pairs from a cell were quantified, and the average is indicated with a bar for each step. A.U: arbitrary units. The mean is indicated with a bar. *P < 0.05; **P < 0.005; ***P < 0.0005 (Mann–Whitney *U* test).

Human kinetochores are composed of more than 100 kinds of molecules, although many of them reside only transiently on kinetochores during mitosis^2^. These include components of the SAC, as well as microtubule-associated proteins such as CLIP-170, dynein, and Ska1. Other proteins, like CENP-E and CENP-F, localize to kinetochores throughout mitosis, but in considerably decreased amounts after metaphase. To get insight into the molecular requirement in each phase of the process of chromosome alignment, we observed the localization of various molecules on kinetochores. Firstly, we confirmed that Hec1, KNL1, and Zwint-1, components of the KMN (KNL1–Mis12–Ndc80) network^21^, stably localize to kinetochores during prometaphase (Fig. 3A,B). For transient kinetochore components, we found that there are three localization patterns during prometaphase. Spindly, BubR1, and Zw10 showed the highest kinetochore localization from NEBD to the prometaphase rosette, and decreased thereafter (Fig. 3A,B). In contrast, kinetochore localization of CLIP-170, CENP-E, Nde1, and dynein intermediate chain (DIC) was low at NEBD, then increased in the prometaphase rosette, and decreased again thereafter (Fig. 3A,B). In contrast to these two groups, Ska1 localization to kinetochores gradually increased and peaked in the equatorial ring (Fig. 3A,B), as reported previously^22^. Collectively, we found that transient kinetochore components enriched in prometaphase show different localization patterns in each phase of chromosome alignment, but many of them show a peak of kinetochore localization in the prometaphase rosette. Considering that most of the kinetochores form lateral attachments in the prometaphase rosette, molecules involved in lateral attachment are likely to be among these kinetochore components enriched in the prometaphase rosette.

### Regulation of transient kinetochore components in prometaphase by Aurora B

**Figure 4:**
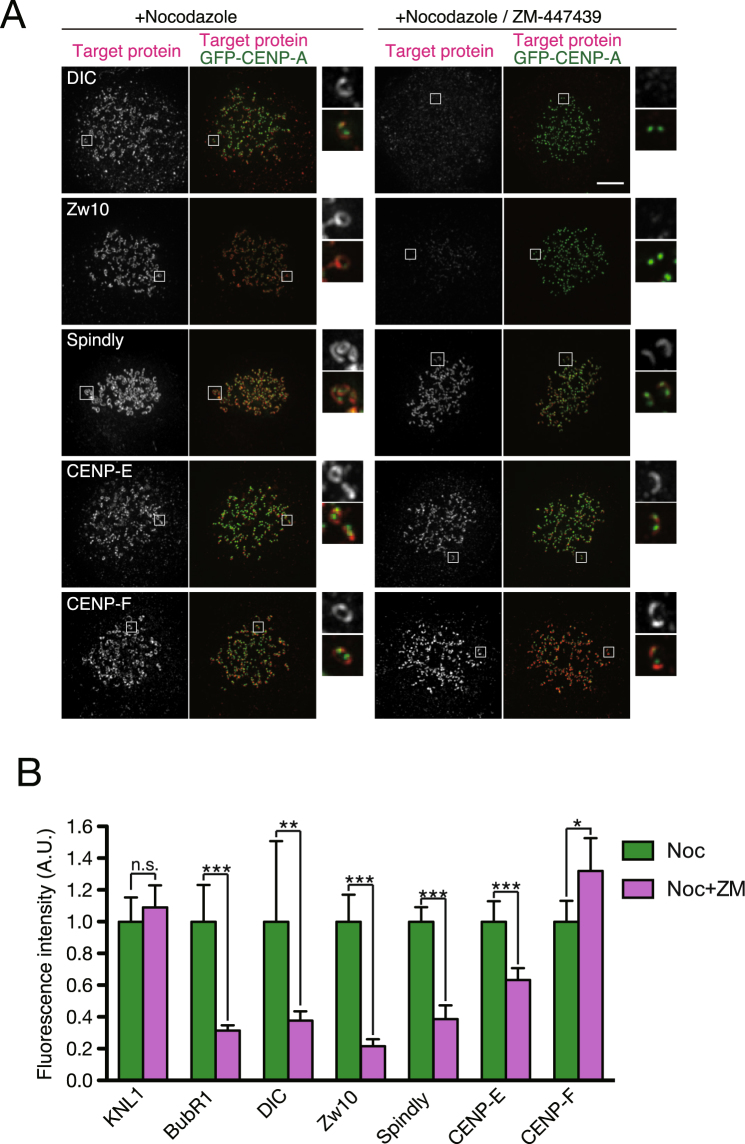
Effect of Aurora B inhibition on the localization of kinetochore components. (**A**) Kinetochore localization of kinetochore components in nocodazole-treated cells in the presence or absence of an Aurora B inhibitor. HeLa cells expressing EGFP–CENP-A (green) were treated with nocodazole in the presence or absence of ZM-447439, and immunostained with an antibody against each kinetochore component (red), as indicated. A magnified view of a kinetochore pair boxed in the panel is shown in insets. Scale bar: 5 μm. (**B**) Signal intensity of kinetochore components in nocodazole-treated cells in the presence (Noc + ZM) or absence (Noc) of an Aurora B inhibitor. Average signal intensity in the absence of ZM-447439 was set as 1 for each kinetochore component. Fluorescent intensity was quantified from 10 cells for each condition. Representative data from three independent experiments are shown. A.U: arbitrary units. Error bars represent S.D. *P < 0.05; **P < 0.005; ***P < 0.0005 (Student’s *t*-test). n.s., not statistically significant.

Aurora B is a mitotic kinase that plays a crucial role in the correction of erroneous kinetochore microtubule attachments^23^. Aurora B also plays a role in the kinetochore localization of the SAC components by inhibiting KNL1 binding of PP1, which dephosphorylates the MELT repeats of KNL1 to release the SAC components^24,25^. Aurora B phosphorylates kinetochore substrates during prometaphase, before end-on attachment is formed, when kinetochores are not under tension and substrates are closer to the inner centromere where Aurora B is enriched^26^. As many transient kinetochore components localize to kinetochores during prometaphase, we examined the role of Aurora B on the localization of these transient kinetochore components. We compared their kinetochore localization in nocodazole-treated cells with or without ZM-447439, an Aurora B inhibitor. As shown in Fig. 4A and B, kinetochore localization of BubR1, a SAC component, decreased as reported previously^27–29^. Kinetochore localization of DIC, Zw10, and Spindly also markedly decreased when Aurora B activity was inhibited. CENP-E localization to kinetochores decreased as well, to a lesser extent (Fig. 4A,B). In contrast, localization of KNL1 and CENP-F to kinetochores did not markedly decrease (Fig. 4A,B). These results suggest that Aurora B activity is required for kinetochore localization of some, but not all, of the transient components enriched during prometaphase.

### Formation of the prometaphase rosette depends on dynein and microtubule depolymerization

**Figure 5:**
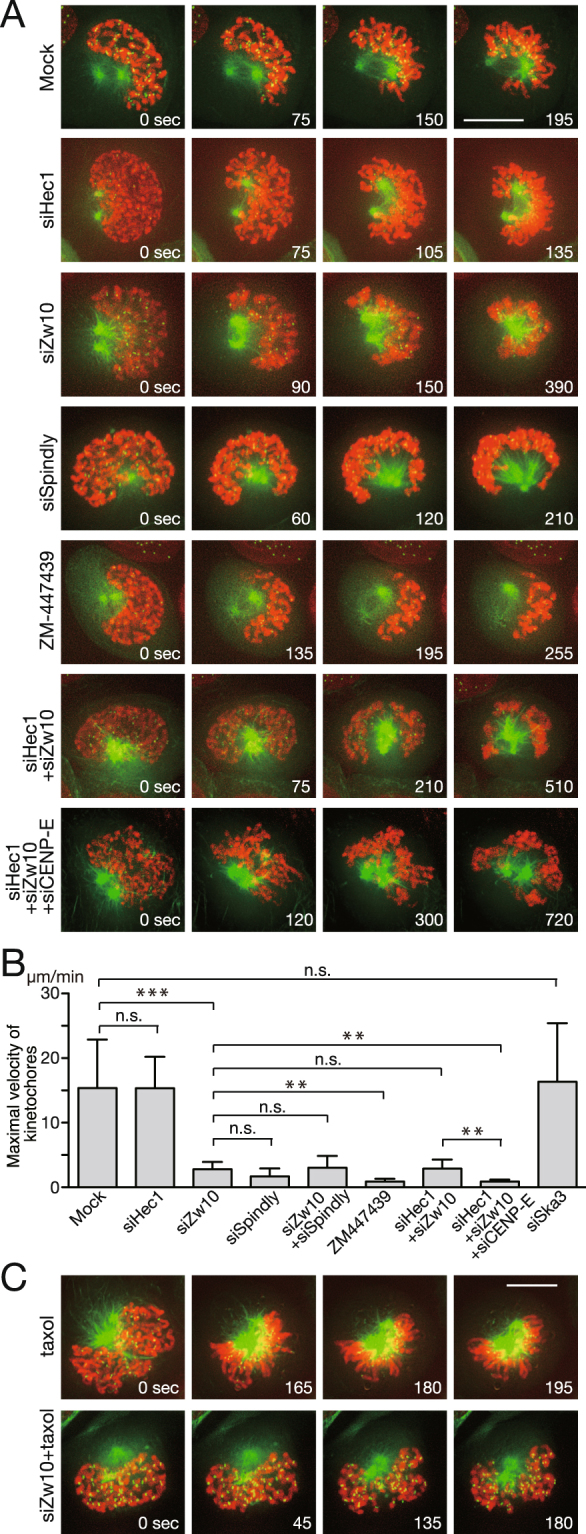
Dynein is responsible for the rapid kinetochore motion in the formation of the prometaphase rosette. (**A**) Kinetochore motion in cells soon after NEBD. HeLa cells expressing EGFP–α-tubulin (green), EGFP–CENP-A (green), and H2B–mCherry (red) were imaged at 15 s intervals, starting from NEBD. Scale bar: 10 μm. (**B**) Maximal velocity of kinetochores in cells soon after NEBD. HeLa cells expressing EGFP–CENP-A and EGFP–α-tubulin was imaged at 550 ms intervals after NEBD, and the motion of kinetochores are tracked to calculate the kinetochore velocity, which is shown in the graph. At least 14 kinetochores from 3 cells were tracked for each condition. Representative data from three independent experiments are shown. Error bars represent S.D. **P < 0.005; ***P < 0.0005 (Student’s *t*-test). n.s., not statistically significant. (**C**) Kinetochore motion in cells soon after NEBD in the presence of taxol. Cells were observed as in (**A**). Scale bar: 10 μm.

**Figure 6:**
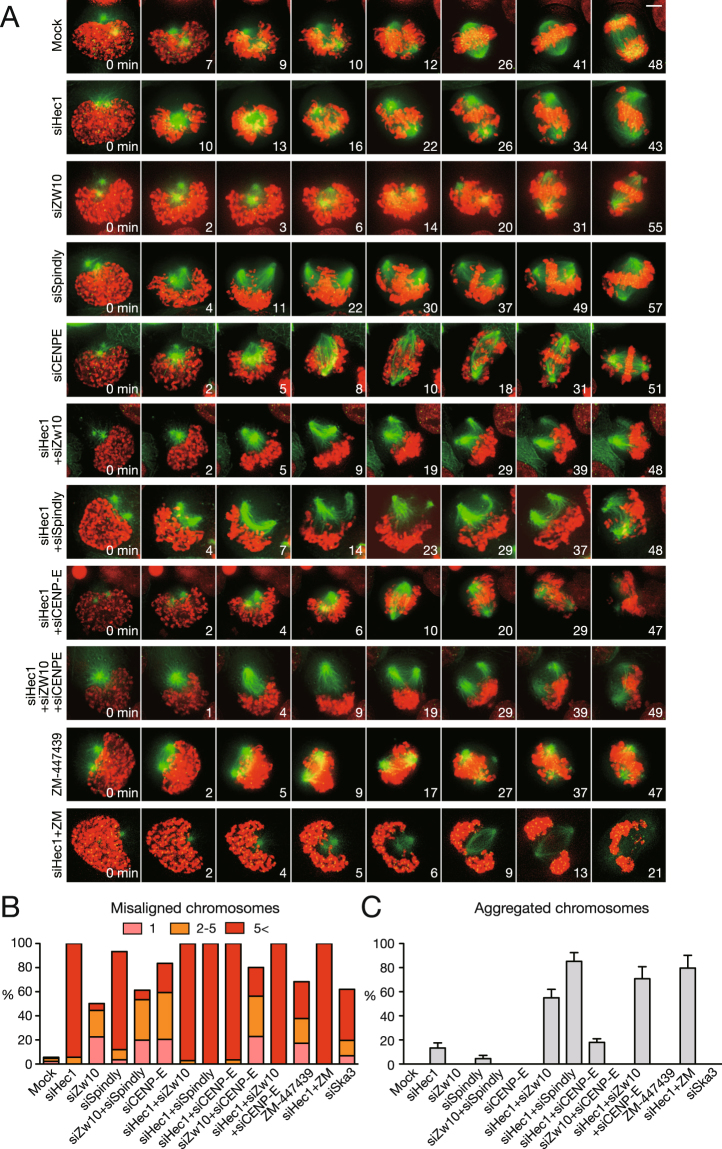
Simultaneous depletion of molecules involved in lateral and end-on attachment results in severe attachment defects of kinetochores to microtubules. (**A**) Defects in chromosome alignment in cells depleted of molecules involved in kinetochore–microtubule attachment. HeLa cells expressing EGFP–α-tubulin (green), EGFP–CENP-A (green), and H2B–mCherry (red) were imaged at 1 min intervals, starting from NEBD. Scale bar: 5 μm. (**B**) Proportion of cells with misaligned chromosomes when they were depleted of molecules involved in kinetochore–microtubule attachment. HeLa cells expressing EGFP–CENP-A were treated with MG132 for 1 h, stained for α-tubulin and chromosomes, and mitotic cells were observed to count the number of misaligned chromosomes. At least 100 cells were observed for each condition. Representative data from three independent experiments are shown. (**C**) Rate of cells with aggregated chromosomes treated as in (**A**). At least 100 cells were observed for each condition. Representative data from three independent experiments are shown. Error bars represent S.D.

How is the prometaphase rosette formed? To answer this question, we observed the formation of the prometaphase rosette in HeLa cells expressing EGFP–α-tubulin, EGFP–CENP-A, and H2B–mCherry and tracked kinetochore motion. As shown in Fig. 5A, kinetochores rapidly moved towards the surface of the nascent spindle after NEBD (Fig. 5A, Mock, Supplementary Movie 3). Intriguingly, the prometaphase rosette was formed in cells depleted of Hec1, which is required for end-on attachment, following rapid kinetochore motion, consistent with the notion that lateral attachment is predominant in the prometaphase rosette and Hec1 is dispensable for lateral attachment (Fig. 5A, siHec1, Supplementary Movie 4)^6–8^. Extensive depletion of Hec1 was assured by the inability to maintain the SAC in the presence of misaligned kinetochores (see Fig. 6A, siHec1)^8^. It was previously shown that dynein is responsible for the rapid motion of kinetochores attaching to the lateral surface of microtubules. Therefore, we observed cells depleted of Zw10, a component of the RZZ complex required for kinetochore localization of dynein^30^. In Zw10-depleted cells, rapid kinetochore motion was not seen but, interestingly, kinetochores still moved slowly towards the spindle surface, forming a chromosome configuration similar to the prometaphase rosette (Fig. 5A, siZw10, Supplementary Movie 5). Similar chromosome motion was also seen in cells depleted of Spindly, which is also required for kinetochore localization of dynein (Fig. 5A, siSpindly, Supplementary Movie 6)^31^. In contrast, in the presence of ZM-447439, chromosomes gathered at one side of the spindle were expelled from the spindle (Fig. 5A, ZM-447439, Supplementary Movie 7), suggesting that lateral attachment was not formed in Aurora B-inhibited cells, probably because of the decrease in the kinetochore components required for lateral attachment including not only dynein, but also CENP-E (Fig. 4, see below). Microtubule destabilization by MCAK activity, which is suppressed when phosphorylated by Aurora B^32–34^, may also contribute to defective rosette formation in ZM-447439-treated cells due to reduced kinetochore capture by microtubules. Chromosome motion away from the spindle pole is supposedly due to polar ejection forces created by chromokinesins such as Kid (see below).

We wondered how chromosomes assemble around the spindle in Zw10- or Spindly-depleted cells without rapid motion driven by dynein. One possibility is that end-on attachment is directly formed, and end-on-attached chromosomes move towards the spindle surface by microtubule depolymerization. To verify this, we observed cells depleted of both Zw10 and Hec1. As shown in Fig. 5A, kinetochores still moved to the spindle surface without rapid motion, suggesting that end-on attachment is not involved in the chromosome motion (Fig. 5A, siHec1 + siZw10, Supplementary Movie 8). Another possibility is that lateral attachment is maintained at microtubule ends, which tethers chromosomes during microtubule depolymerization. A candidate kinetochore-tethering molecule at microtubule ends is CENP-E, which has been reported to track depolymerizing microtubule ends^35,36^. Therefore, we depleted CENP-E together with Zw10 and Hec1 and observed chromosome motion after NEBD. Chromosome motion to the spindle surface did not occur in this case (Fig. 5A, siHec1 + siZw10 + siCENP-E, Supplementary Movie 9), supporting the idea that chromosomes are tethered to microtubule ends by CENP-E and moved by microtubule depolymerization. Efficiency of RNAi for each molecule was verified by immunoblotting (Supplementary Fig. S2A). When we observed kinetochore–microtubule attachment by immunofluorescence staining, the majority of kinetochores were not attached to microtubules in cells depleted of the three molecules (Supplementary Fig. S2B).

These results were further confirmed by measuring the velocity of kinetochore movement to the spindle surface. Kinetochore speed estimated by tracking data from live-cell imaging of mock-treated cells taken at 15 s intervals was much slower than the reported speed of dynein-driven kinetochore motion (data not shown). But when we tracked kinetochores at 550 msec intervals, we found that kinetochores did not move at a constant speed, but transiently moved at a high speed (15.37 ± 7.51 (mean ± S.D.) μm/min; n = 16) that is comparable to the reported kinetochore speed driven by dynein (Fig. 5B, Supplementary Fig. S3A,B)^37^. It is noteworthy that rapid kinetochore motion ceased when kinetochores reached the spindle surface, and kinetochores did not move poleward further before changing direction towards the spindle equator (Fig. 5A, Supplementary Fig. S3B). The high kinetochore speed seen in control cells did not change in Hec1-depleted cells, consistent with the idea that Hec1 is dispensable for lateral attachment (15.34 ± 4.86 μm/min; n = 13, Fig. 5B, Supplementary Fig. S3B). In contrast, kinetochore speed was markedly dampened in Zw10- or Spindly-depleted cells (2.79 ± 1.13 μm/min; n = 15 (siZw10), 1.68 ± 1.25 μm/min; n = 14 (siSpindly), Fig. 5B, Supplementary Fig. S3B), corroborating that dynein is responsible for the rapid kinetochore motion, although kinetochores still moved but at a lower speed. Codepletion of Zw10 and Spindly did not reduce kinetochore speed further (3.01 ± 1.86 μm/min; n = 17, Fig. 5B). In cells depleted of Hec1 and Zw10, kinetochores moved at a low speed comparable to that in Zw10-depleted cells (2.89 ± 1.39 μm/min; n = 15, Fig. 5B, Supplementary Fig. S3B), excluding the possibility that end-on attachment is responsible for kinetochore motion in the absence of dynein. In contrast, when CENP-E was depleted together with Hec1 and Zw10, kinetochore speed was further reduced to almost zero (0.90 ± 0.30 μm/min; n = 17, Fig. 5B, Supplementary Fig. S3B), showing that CENP-E is responsible for the slower kinetochore motion. CENP-E depletion, either alone or with Hec1 depletion, did not alter rapid kinetochore motion, but kinetochore speed was markedly reduced when CENP-E was codepleted with Zw10 (Supplementary Fig. S3C). Cells treated with ZM-447439 also showed markedly reduced kinetochore speed comparable to that in cells depleted of Hec1, Zw10, and CENP-E (0.88 ± 0.45 μm/min; n = 13, Fig. 5B). In cells depleted of Ska3, a component of the Ska complex like Ska1, the prometaphase rosette was formed with kinetochore speed comparable to that of mock-treated cells (16.34 ± 9.08 μm/min; n = 19, Fig. 5B and data not shown), consistent with the notion that the Ska complex plays a role in end-on attachment, but not in lateral attachment^38–40^. We also examined the effect of polar ejection force driven by Kid, a kinesin-10 motor that localizes to chromosome arms^41^, on poleward kinetochore motion. Although Kid depletion did not significantly increase kinetochore speed, the reduced kinetochore speed caused by Spindly depletion was partially restored by Kid depletion (Supplementary Fig. S3D,E), suggesting that polar ejection force by Kid counteracts poleward kinetochore motion^10^. When we compared the size of the prometaphase rosette in Kid-depleted cells by the distance of kinetochores from the center of the spindle poles, it was smaller than that in mock-treated cells (Supplementary Fig. S3F), showing that some of the chromosomes are inside of the nascent spindle, which is in agreement with the previous report^7^. To corroborate the finding that microtubule depolymerization is responsible for chromosome motion in the absence of dynein, we tested whether chromosome motion to the spindle surface is inhibited in Zw10-depleted cells when microtubule dynamics are suppressed by taxol treatment. Indeed, chromosomes did not move to the spindle surface (Fig. 5C, siZw10 + taxol, Supplementary Movie 11), confirming that chromosomes move by microtubule depolymerization in the absence of rapid motion driven by dynein. In control cells treated with taxol alone, we found that the prometaphase rosette was formed normally (Fig. 5C, taxol, Supplementary Movie 10). Taken together, we conclude that rapid kinetochore motion during the formation of the prometaphase rosette is driven by dynein, but kinetochores move to the spindle surface even in the absence of dynein at a lower speed by being tethered to the lateral surface of depolymerizing microtubule ends.

### Lateral attachment facilitates chromosome alignment and bi-orientation establishment

Lateral attachment is involved not only in the rapid poleward motion soon after NEBD but also in chromosome congression along the spindle surface driven by CENP-E and the polar ejection force, which is mediated by chromokinesins such as Kid^5–8^. It has been suggested that lateral attachment facilitates the establishment of bi-orientation^7,9^, although the significance of lateral attachment in bi-orientation establishment has not been experimentally demonstrated. Therefore, we observed chromosome alignment and bi-orientation establishment in cells depleted of molecules involved in lateral and/or end-on attachment. HeLa cells expressing EGFP–α-tubulin, EGFP–CENP-A, and H2B–mCherry were observed by live-cell imaging throughout mitosis. In mock-treated cells, chromosomes aligned efficiently to the metaphase plate (Fig. 6A Mock, 41 min; Supplementary Movie 12) and segregated properly in anaphase (Fig. 6A Mock, 48 min). In Hec1-depleted cells, prometaphase rosettes were formed (Fig. 6A siHec1, 13 min; Supplementary Movie 13) as shown before. Chromosomes were then partially assembled to the spindle equator (Fig. 6A siHec1, 26–43 min), but massively missegregated when cells slipped into anaphase due to SAC deficiency, as we have recently reported (Supplementary Movie 13)^8^. In cells depleted of Zw10, the metaphase plate was formed, but many chromosomes did not align to it (Fig. 6A,B siZw10, 55 min; Supplementary Movie 14), as reported previously^4^. A similar defect was seen in Spindly-depleted cells (Fig. 6A,B siSpindly, Supplementary Movie 15), but more severe than that in Zw10-depleted cells, as reported previously, implicating the RZZ complex in suppression of end-on attachment^42^. Cells codepleted of Zw10 and Spindly showed similar level of chromosome misalignment compared to Zw10-depleted cells (Fig. 6B siZw10 + siSpindly). The chromosome alignment defect in Spindly-depleted cells was partially rescued by co-depleting Kid, suggesting that loss of polar ejection force facilitated chromosome alignment through ameliorated kinetochore accumulation to the spindle surface (Supplementary Fig. S4A). CENP-E-depleted cells also exhibited chromosome misalignment (Fig. 6A siCENP-E, 51 min; Supplementary Movie 16), as already shown^43–45^. Intriguingly, when Hec1 and Zw10 were simultaneously depleted, chromosomes were excluded from the spindle, forming an aggregate (Fig. 6A siHec1 + siZw10, 48 min; Supplementary Movie 17). The appearance of the chromosomes in cells depleted of Hec1 and Zw10 is distinct from that of Hec1-depleted cells. In Hec1-depleted cells, most chromosomes are incorporated into the spindle, maintaining the lateral attachment of kinetochores to microtubules until cells slip into anaphase, as reported previously^6,8^, which was confirmed by immunofluorescence staining (Supplementary Fig. S4B siHec1). In contrast, kinetochore attachments to microtubules were mostly lost in Hec1, Zw10-codepleted cells (Supplementary Fig. S4B siHec1 + siZw10). Aggregated chromosomes were seen in more than half of the mitotic cells depleted of Hec1 and Zw10, but in only around ten percent of Hec1-depleted cells, although other chromosome misalignment was seen in most Hec1-depleted cells (Fig. 6B,C). Similar findings were seen in cells depleted of Hec1 and Spindly (Fig. 6A–C siHec1 + siSpindly, Supplementary Movie 18). Aggregated chromosomes were not apparent in Zw10-, or Spindly-depleted cells as well as in Zw10, Spindly-codepleted cells (Fig. 6C). These findings suggest that chromosome aggregation is the result of defects in both lateral and end-on attachment, which was mentioned previously^46^. When Hec1 and CENP-E were depleted simultaneously, the proportion of cells showing aggregated chromosomes was relatively low, although chromosomes were massively missegregated (Fig. 6A–C siHec1 + siCENP-E; Supplementary Movie 19). In cells codepleted of Zw10 and CENP-E, most of the cells showed misaligned chromosomes, but no aggregated chromosomes were seen (Fig. 6B,C siZw10 + siCENP-E). When CENP-E and Zw10 were depleted together with Hec1, the proportion of cells with aggregated chromosomes further increased compared to that in Hec1, Zw10-codepleted cells (Fig. 6A–C siHec1 + siZw10 + siCENP-E; Supplementary Movie 20). Kinetochore attachments to microtubules were severely compromised in these cells (Supplementary Fig. S4B siHec1 + siZw10 + siCENP-E). These data suggest that both dynein and CENP-E play a role in maintaining kinetochore–microtubule attachment in the absence of end-on attachment, although the contribution of CENP-E is smaller than that of dynein. In cells depleted of Ska3, misaligned chromosomes were seen in more than half of the cells, but no aggregated chromosomes were seen (Fig. 6B,C siSka3). We also examined the role of Aurora B in bi-orientation establishment. In cells treated with ZM-447439, many chromosomes were misaligned, as is already known^27^, partly due to defects in the correction of erroneous attachments, while aggregated chromosomes were not seen (Fig. 6A–C ZM-447439; Supplementary Movie 21). But when Aurora B was inhibited in Hec1-depleted cells, aggregated chromosomes were frequently seen, implying that Aurora B functions in the maintenance of kinetochore–microtubule attachment by regulating lateral attachment (Fig. 6A–C siHec1 + ZM; Supplementary Movie 22). In summary, defects in both lateral and end-on attachment cause total loss of kinetochore–microtubule attachments, resulting in aggregation of chromosomes, indicating that lateral attachment contributes to the establishment of bi-orientation.

## Discussion

**Figure 7:**
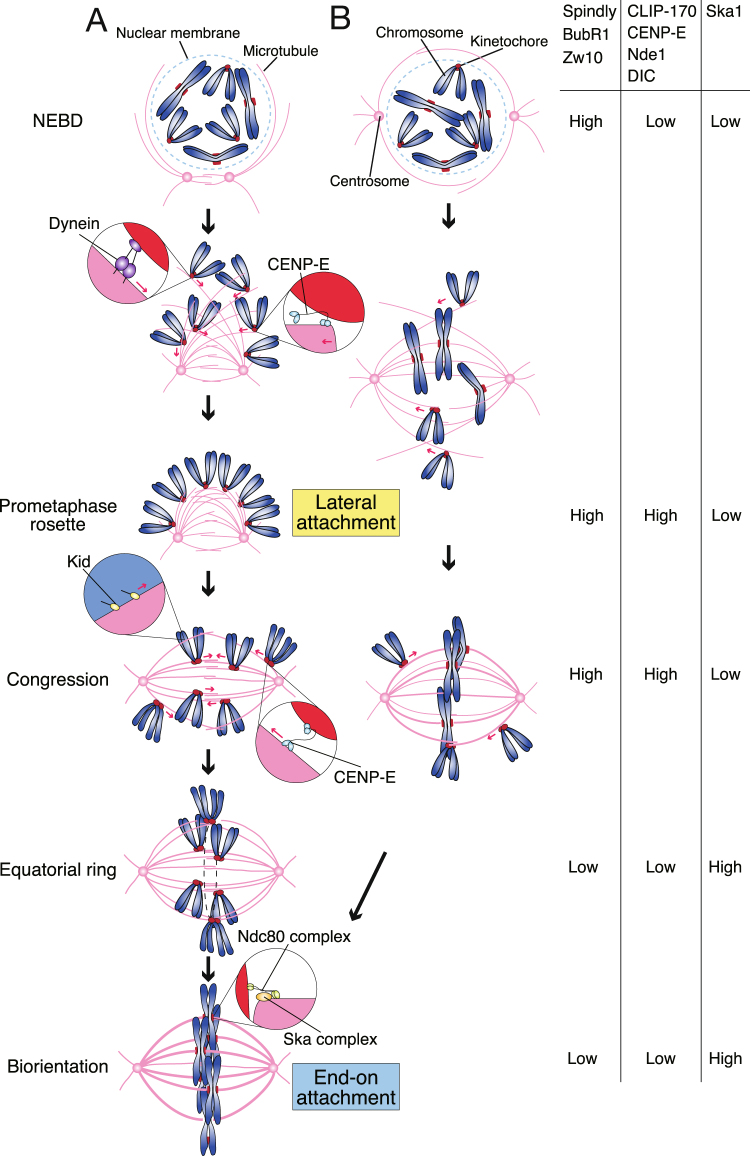
Kinetochore attachments to microtubules and chromosome motion in the process of bi-orientation establishment. Schematic diagram showing the situation where (**A**) the axis connecting centrosomes are outside of chromosome mass, or (**B**) centrosomes are at opposite sides of the nucleus at NEBD. In (**A**), kinetochores are initially captured by the lateral surface of microtubules, and transported towards spindle poles by dynein, forming the prometaphase rosette. CENP-E also contributes to the poleward motion of the kinetochores, supposedly through tethering kinetochores at depolymerizing microtubule ends. Laterally-attached kinetochores are then moved towards the spindle equator along microtubules (congression). CENP-E and Kid are involved in chromosome congression. Before bi-orientation is established, laterally-attached kinetochores align at the circumference of the spindle equator, referred to as the equatorial ring. Lateral attachment is then converted to end-on attachment at the spindle equator when bi-orientation is established. The Ndc80 complex plays a major role in end-on attachment, which is stabilized by the Ska complex. Red arrows indicate motion of chromosomes forming lateral attachment. Relative kinetochore localization of kinetochore components in each step is shown on the right. In (**B**), end-on attachment is established for a fraction of kinetochores locating inside the nascent spindle soon after NEBD. Therefore, typical prometaphase rosette and equatorial ring are not seen, but transition from lateral to end-on attachment are still seen for the kinetochores outside of the nascent spindle in a similar manner as in (**A**). See text for details.

In this paper, we clarified how the prometaphase rosette is formed (Fig. 7A). A typical prometaphase rosette is formed when the axis connecting centrosomes is outside of the chromosome mass. Microtubules growing from centrosomes elongate towards the chromosome mass, while interdigitating with microtubules from opposite spindle poles forming the nascent spindle. Overshot microtubules capture chromosomes with their lateral surfaces, and these chromosomes are quickly transported towards the spindle surface by dynein before the prometaphase rosette is formed (Figs 5 and 7A). Our results show that kinetochores in the prometaphase rosette are not under tension, and are positive for Mad2 localization, suggesting that they are uniformly forming lateral attachments (Figs 1 and 2). Therefore, the prometaphase rosette indicates the completion of lateral attachment before end-on attachment is formed. Utilizing this property, we could recognize differences in the temporal localization pattern between kinetochore proteins in early mitosis (Figs 3 and 7) that may represent the structural change of kinetochores. We found that the prometaphase rosette is observed in nearly half of the mitotic events in HeLa cells. One possibility is that the prometaphase rosette is only seen in cells cultured on plates, on which cells are flattened, potentially causing chromosome scattering outside the spindle^47^. However, prometaphase rosettes are also seen in subcutaneous tumors derived from HeLa cells transplanted into SCID (severe combined immunodeficiency) mice (Supplementary Fig. S5A). We also found that prometaphase rosettes were frequently observed in mitotic HeLa cells cultured in suspension (data not shown). We further confirmed that the prometaphase rosette is seen in tissue samples of human gastric cancer cells (Supplementary Fig. S5B). These examples exclude the possibility that the prometaphase rosette is only seen in cells cultured on plates. Even when centrosomes are at opposite sides of the nucleus at NEBD, chromosomes initially outside of the nascent spindle attach laterally to the spindle surface, and then move towards the spindle equator along spindle surface (Figs 1A–ii and 7B). Therefore, we propose that lateral attachment is not a backup mechanism for kinetochores that have failed to directly form end-on attachment, but a regular mechanism to ensure incorporation of chromosomes to the spindle for bi-orientation establishment.

The molecular basis of lateral attachment is not well understood compared to that of end-on attachment. By dividing prometaphase into several distinct steps, including the formation of the prometaphase rosette, we found that transient kinetochore components show dynamic localization patterns even within prometaphase (Figs 3 and 7). Many of these transient kinetochore components are enriched on kinetochores in the prometaphase rosette (Figs 3 and 7). These include the microtubule-interacting proteins (CLIP-170 and CENP-E) as well as dynein-related proteins (Spindly, NDE1, DIC), RZZ complex components (Zw10), and SAC components (BubR1). Considering that kinetochores form lateral microtubule attachments in the prometaphase rosette, molecules involved in lateral attachment are supposedly included among them. These molecules overlap with components of the expandable module on unattached kinetochores reported by Wynne and Funabiki^28,29^. Among these, the RZZ complex was recently suggested to form higher-order oligomers that may be a molecular basis for the fibrous corona, a kinetochore structure visible only before microtubule attachment^48^. Two opposing motors, dynein and CENP-E, are obviously working at the interface between kinetochores and microtubules during lateral attachment, judging from their role in chromosome motion along microtubules^3,5^. Our data showing that kinetochore–microtubule attachment was lost in cells depleted of Hec1, Zw10, and CENP-E in early prometaphase (Supplementary Fig. S2B) strongly suggest that these two motors are involved in the lateral attachment itself, as well as in motility during lateral attachment. It was also reported that CENP-E helps tether kinetochores at microtubule ends when lateral attachment is converted to end-on attachment^36^. There are other molecules potentially involved in lateral attachment, such as CENP-F and CLIP-170, which are enriched on prometaphase kinetochores and can bind to microtubules. It is of note that Zw10 is not only responsible for kinetochore localization of dynein, but also that of CLIP-170 through dynactin^49^. Our recent data suggest that CLIP-170 plays a role in tethering kinetochores to microtubule ends to resist the dynein-mediated poleward force, which is independent of CENP-E^50^, implicating CLIP-170 in lateral attachment.

Dynein may be more critical than CENP-E for lateral attachment, because Hec1, Zw10-codepleted cells show aggregated chromosomes much more frequently than Hec1, CENP-E-codepleted cells (Fig. 6C). It is worth noting that CENP-E works more efficiently on detyrosinated microtubules, which are enriched in stable, bundled microtubules^51^,whereas dynein prefers tyrosinated microtubules, which are commonly seen in unstable single microtubules, for its motility^52,53^. Such properties can explain the sequential chromosome motion along microtubules; first, towards spindle poles by dynein, and then towards the spindle equator by CENP-E^54^. In our observations, kinetochores do not necessarily reach spindle poles, but halt poleward motion on the spindle surface, supposedly through attachment to bundled spindle microtubules (Supplementary Fig. S1B). One explanation for the importance of dynein over CENP-E is that dynein plays a complementary role in supporting kinetochore–microtubule attachment in early prometaphase when both CENP-E and end-on attachment do not work efficiently. Dynein also contributes to efficient kinetochore accumulation on the spindle surface by counteracting the polar ejection force produced by Kid, as reported previously (Supplementary Fig. S3D–F)^10^. Nevertheless, our data suggest that CENP-E also plays a role in early prometaphase to form rosette-like structures in the absence of dynein, supposedly through tethering kinetochores at depolymerizing microtubules ends (Figs 5 and 7)^35^.

Collective information from recent studies indicates that localization of most of the transient kinetochore components enriched in prometaphase is dependent on KNL1^55,56^, and Bub1 acts as their platform by binding to the MELT repeats on KNL1 phosphorylated by Mps1 and Plk1^57–62^. We found that many transient kinetochore components depend on Aurora B activity for kinetochore localization (Fig. 4). One plausible mechanism is by facilitating Mps1 kinetochore localization^63,64^, which binds to the N-terminal region of Hec1^65–67^. Another mechanism is to inhibit KNL1 binding of PP1, which dephosphorylates the MELT repeats of KNL1, by phosphorylating the PP1 binding site^24,25^. It was reported that Aurora B is involved in expansion of unattached kinetochores^28^. Our findings, and those of Wynne *et al*., represent a further layer of regulation by Aurora B on kinetochore–microtubule attachment, which was recently proposed to be involved in conversion from lateral to end-on attachment^68^.

As the SAC and lateral attachment are supposed to be dependent on these transient kinetochore components, it is plausible that KNL1 is organizing these two activities. In contrast, the Ndc80 complex is responsible for the end-on attachment, with other molecules like the Ska complex^22,38,40,69,70^ (Fig. 7). It was recently shown that Mps1 exclusion from kinetochores by microtubule attachment to Hec1 links kinetochore–microtubule attachment with the SAC silencing^63,64^, which also results in the removal of the lateral attachment module. The relationship between lateral attachment and end-on attachment may be bi-directional; in *C. elegans*, the RZZ complex suppresses the end-on attachment by binding to Hec1^71,72^. Suppression of end-on attachment by the RZZ complex has also been suggested in human cells^42,73^. These data imply that lateral attachment is not only an unstable intermediate in the formation of stable end-on attachment, but is beneficial in prometaphase when early establishment of end-on attachment is rather unfavorable for correction of erroneous attachments. It was proposed that when spindle poles are close to each other, a single kinetochore can be captured by microtubules from both spindle poles, leading to the formation of merotelic attachments and increased rates of chromosome missegregation^74,75^. In this respect, it would be advantageous to form lateral rather than end-on attachments, to avoid merotelic attachments in the prometaphase rosette when the spindle poles are at one side of the chromosome mass.

Cancer cells frequently show increased rates of chromosome missegregation, which is referred to as chromosomal instability (CIN). Defects in the mechanisms of chromosome segregation are supposed to cause CIN, but the defects should be within a range permissive for cell survival. In contrast to the mechanism for end-on attachment that is essential, defects in lateral attachment are likely to be tolerable, but still affect fidelity of chromosome segregation. Further study of lateral attachment will thus contribute to the elucidation of the underlying cause of CIN.

## Materials and Methods

### Cell culture, synchronization, drug treatment and transfection

HeLa Kyoto cells, which have been tested negative for mycoplasma contamination, were cultured in DMEM supplemented with 10% fetal bovine serum (FBS). To synchronize at early mitotic phase, cells were cultured in the presence of 2 mM thymidine for 24 h, released from thymidine for 10 h, and then fixed and stained. Monastrol (Enzo Life Science) was used at 100 μM for 2 h to observe monopolar spindles. To arrest cells at mitosis, 1 μM nocodazole (Sigma) was added 2 h before fixation. Taxol (Wako) was used at 20 nM for 3 h to suppress microtubule depolymerization. For inhibition of Aurora B kinase activity, ZM-447439 (Tocris Bioscience) was used at 2 μM for 2 h together with 10 μM MG132 (Sigma) in the presence of 1 μM nocodazole. Transfection of siRNA oligonucleotides was carried out by incubating 100 nM duplexed siRNA with RNAi MAX (Invitrogen) for 48 hours in antibiotic-free growth medium.

### Antibodies

The following antibodies were commercially purchased and used at the indicated dilutions for immunofluorescence (IF) and immunoblotting (IB); Actin (I-19, Santa Cruz Biotechnology, IB; 1/2000), α-tubulin (B-5-1-2, Sigma, IF; 1/1000), BubR1 (Bethyl Laboratories, IF; 1/500), CENP-E (Sigma, IF; 1/500, IB; 1/2000), CENP-F (Abcam, IF; 1/1000), CLIP170 (Santa Cruz Biotechnology, IF; 1/500), dynein intermediate chain (DIC, Sigma, IF; 1/500), Hec1 (9G3, Abcam, IF; 1/1000, IB; 1/2000), Kid (Cytoskeleton Inc., IB: 1/1000), KNL1 (Novus Biologicals, IF; 1/500), Mad2 (Novus Biologicals, IF; 1/500), Nde1 (Protein Tech Group, IF; 1/500), Ska1 (Abcam, IF; 1/500), Spindly (Bethyl Laboratories, IF; 1/500, IB; 1/2000), Zwint-1 (Bethyl Laboratories, IF; 1/500), Zw10 (Cosmo Bio, IF; 1/200, IB; 1/2000), pericentrin (Abcam, IF; 1/500).

### RNAi

The synthetic oligonucleotides targeting human Hec1 and CENP-E for RNAi were obtained from Invitrogen (Stealth). The sequences were as follows; Hec1 (5′-UCAGCCAUUCUUGACCAGAAAUUAA-3′), and CENP-E (5′-CGGCUCAAGGAAGGCUGUAAUAUAA-3′). The siRNA sequences targeting for Zw10, Spindly and Kid were obtained from JBioS. The sequences were as follows: Zw10 (5′-UGAUCAAUGUGCUGUUCAATT-3′), Spindly (5′-GAAAGGGUCUCAAACUGAATT-3′), and Kid (5′-AAGAUUGGAGCUACUCGUCGUTT-3′). The sequences of mixed siRNAs targeting for Ska3, obtained from ThermoFisher Scientific (ON-TARGETplus), were as follows: 5′-GGAAGAGCCCGUAAUUGUA-3′, 5′-GAUCGUACUUCGUUGGUUU-3′, 5′-AAUCCAGGCUCAAUGAUAA-3′, and 5′-CAUCGUAUCCCAAGUUCUA-3′.

### Live-cell imaging

HeLa cells expressing EGFP–α-tubulin, EGFP–CENP-A, and H2B–mCherry were grown in glass chambers (Thermo). One hour before imaging, the medium was changed to pre-warmed Leibovitz’s L-15 medium (Life Technologies) supplemented with 20% FBS and 20 mM HEPES, pH 7.0. Recordings were made in a temperature-controlled incubator at 37 °C. All time-lapse images were collected with an Olympus IX-71 inverted microscope (Olympus) controlled by DeltaVision softWoRx (Applied Precision) using a 100 × 1.40 NA Plan Apochromat oil objective lens (Olympus). For measurement of maximal kinetochore velocity, Z-series of 3 sections in 0.3 μm increments were captured every 550 msec and image stacks were projected. The value of x–y axes from kinetochore position was tracked with the Manual Tracking plug-in for ImageJ. The velocity was calculated for the maximal constant motion towards the spindle pole.

### Immunofluorescence analysis

For Mad2 staining, cells expressing EGFP–CENP-A were grown on a glass coverslip and fixed with methanol/acetone [1:1] at −20 °C for 10 min. For visualization of BubR1, CLIP-170, Hec1, KNL1, Nde1, Ska1, Spindly and Zwint-1, cells were pre-extracted with PHEM buffer pH 7.0 (60 mM PIPES, 25 mM HEPES, 10 mM EGTA and 2 mM MgSO_4_) containing 0.1% Triton X-100, and fixed with 3.6% formaldehyde in this buffer at 37 °C for 10 min. For CENP-E, CENP-F, dynein intermediate chain and Zw10 staining, cells pre-extracted with 0.1% Triton X-100 in PHEM buffer pH 7.0 were fixed with methanol at −20 °C for 10 min. For pericentrin staining, cells were fixed with 4% paraformaldehyde at 37 °C for 15 min. For visualization of microtubules and kinetochores, cells were fixed as described previously^76^. Cells were fixed with 1% glutaraldehyde in PHEM buffer for 15 min, and quenched with 0.1 g ml^−1^ NaBH_4_ in PHEM for 10 min. Glass coverslips were blocked with 3% BSA in PBS containing 0.01% Triton X-100 (wash buffer) at RT for 30 min, and incubated with primary antibodies overnight at 4 °C and then secondary antibodies at RT for 1 h. Goat anti-rabbit IgG Alexa Fluor-488 and 568, goat anti-mouse Alexa Fluor-488 and 568 (Molecular Probes) were used as secondary antibodies. Fluorescence images were acquired using Olympus IX-71 inverted microscope controlled by DeltaVision softWoRx using a 100 × 1.40 NA Plan Apochromat oil objective lens (Olympus). A series of Z-stacking images obtained at 0.2 μm intervals were deconvoluted using an algorithm with default settings and represented as maximum intensity projections. To measure distances between Hec1 or CENP-A pairs in a series of Z-stacking images acquired at 0.1 μm intervals, intensity-weighted centroid of Hec1 or CENP-A signal were determined using surface tool of Imaris software (version 8.2.0; Bitplane). Intra-kinetochore distance was calculated by dividing the difference between Hec1 pair distance and CENP-A pair distance into two. Fluorescence images in Supplementary Fig. S3F were obtained with a confocal microscope system, LSM 510 META microscope (Carl Zeiss) equipped with a 100 × 1.40 NA Plan Apochromat oil objective lens (Olympus). For excitation of GFP, TRITC, and DAPI, an argon laser (488 nm line), HeNe laser (543 nm line), and Blue Diode (405 nm line) were used, respectively. Z-stacking images in optical sections were obtained with scanning up at 0.48 μm intervals. For measurements of the distance between CENP-A signal and the midpoint of pericentrin signals, their coordinates were analyzed with Fiji software (http://fiji.sc), and distance was calculated by Microsoft Excel.

### Correlative light and electron microscopy (CLEM)

CLEM was performed as described previously^77,78^ with some modifications. In brief, cells were grown on a glass coverslip without grids, and fixed with 3% paraformaldehyde/1% glutaraldehyde in 0.1 M sodium cacodyl buffer for 15 min, then 1% glutaraldehyde in 0.1 M sodium cacodyl buffer for 1 h. Mitotic cells were identified using an Olympus IX-71 inverted microscope (Olympus) controlled by DeltaVision softWoRx (Applied Precision) using UPLSAPO × 100 1.40 numerical aperture (NA) Plan-Apochromat oil objective lens (Olympus). Z-stack optical images (91 focal planes at 0.2 μm intervals) were acquired. To identify the mitotic cells of interest, surrounding cells were removed by scraping. The samples were post-fixed in osmium tetroxide, staining with tannic acid, dehydrated stepwise to 100% ethanol, permeabilized with QY-1 and embedded in EPON. Serial sections (100 nm) of selected mitotic cells were cut using an ultramicrotome, collected on formvar-coated slit mesh grids and post-stained with lead citrate. Serial sections were observed using an electron microscope (H-7600, Hitachi).

### Quantification of fluorescence intensity

Quantification of the signal intensity at kinetochores was conducted with ImageJ. To determine the positions of kinetochores, the circular region encompassing CENP-A signals in each Z-section was defined as region of interest (ROI). The fluorescence intensity of target proteins within the same ROI was measured and background intensity was subtracted. The indicated number of kinetochores in each figure was measured, and results were averaged per cell.

### Western blotting

Cells were lysed in TNE-N (1% NP-40, 100 mM NaCl, 10 mM Tris-HCl, pH 7.5, and 1 mM EDTA) buffer. The protein concentration of cell lysate was measured by Bio-Rad Protein assay kit (Bio-Rad). Cell lysates were boiled for 10 min with 4x NuPAGE LDS sample buffer (Life Technologies) and proteins were separated on NuPAGE SDS-gels (Life Technologies), electroblotted onto a PVDF membrane (Amersham Hybond-P, GE Healthcare), and subjected to immunodetection using appropriate primary antibodies. Blocking and antibody incubations were performed in 3% non-fat milk powder in TBS. Proteins were visualized using horseradish peroxidase-labeled secondary antibodies (Santa Cruz Biotechnology, 1/3,000) and enhanced chemiluminescence, according to the manufacturer’s instructions (GE Healthcare).

### ***In vivo*** tumor transplantation

All animal experimental protocols were approved by the Committee for Ethics of Animal Experimentation, and the experiments were fulfilled according to the guidelines of animal experiments at Akita University. HeLa Kyoto cells (1 × 10^6^ cells) were injected into the subcutaneous tissue of 6-week-old C.B-17 SCID mice (Charles River Laboratories Japan, Inc.). The mice were sacrificed 8 days after the injection. Tumor tissues excised from mice were fixed, and embedded in paraffin.

### Specimens from cancer patients

Gastric adenocarcinoma specimens were obtained from patients who had undergone resection of primary gastric tumors. None of the patients had undergone preoperative radiation or chemotherapy. The study was approved by the ethical review board of Akita University (#1662), and all samples were collected from the surgical pathology files at Akita University Hospital, from between 2008 and 2015, and tissues were obtained with the informed consent of the patients. We confirmed that all methods were performed in accordance with the relevant guidelines and regulations.

### Statistical analysis

Mann–Whitney *U* test was used for comparison of dispersion, and a two-sided Student’s *t* test was used for comparison of average. Samples for analysis in each data set were acquired in the same experiment, and all samples were calculated at the same time for each data set.

### Data availability

All data generated or analyzed during this study are included in this published article and its Supplementary Information files.

## Electronic supplementary material

Supplementary Information

Supplementary Movie 1

Supplementary Movie 2

Supplementary Movie 3

Supplementary Movie 4

Supplementary Movie 5

Supplementary Movie 6

Supplementary Movie 7

Supplementary Movie 8

Supplementary Movie 9

Supplementary Movie 10

Supplementary Movie 11

Supplementary Movie 12

Supplementary Movie 13

Supplementary Movie 14

Supplementary Movie 15

Supplementary Movie 16

Supplementary Movie 17

Supplementary Movie 18

Supplementary Movie 19

Supplementary Movie 20

Supplementary Movie 21

Supplementary Movie 22
