# Author Correction: Lateral attachment of kinetochores to microtubules is enriched in prometaphase rosette and facilitates chromosome alignment and bi-orientation establishment

**DOI:** 10.1038/s41598-018-25175-4

**Published:** 2018-04-30

**Authors:** Go Itoh, Masanori Ikeda, Kenji Iemura, Mohammed Abdullahel Amin, Sei Kuriyama, Masamitsu Tanaka, Natsuki Mizuno, Hiroko Osakada, Tokuko Haraguchi, Kozo Tanaka

**Affiliations:** 1 Department of Molecular Oncology, Institute of Development, Aging and Cancer, Tohoku University, Sendai, 980-8575 Japan; 2 Department of Molecular Medicine and Biochemistry, Akita University Graduate School of Medicine, Akita, 010-8543 Japan; 3 Advanced ICT Research Institute, National Institute of Information and Communications Technology (NICT), Kobe, 651-2492 Japan; 4 Graduate School of Frontier Biosciences, Osaka University, Suita, 565-0871 Japan; 5 Graduate School of Science, Osaka University, Toyonaka, 560-0043 Japan; 6 Present Address: Department of Cell and Molecular Biology, Feinberg School of Medicine, Northwestern University, Chicago, IL 60611 USA

Correction to: *Scientific Reports* 10.1038/s41598-018-22164-5, published online 01 March 2018

The original version of this Article contained a typographical error in the spelling of the author Mohammed Abdullahel Amin, which was incorrectly given as Mohammed Abdhullahel Amin. This has now been corrected in the PDF and HTML versions of the Article.

