# Supplementary material for: Lateral attachment of kinetochores to microtubules is enriched in prometaphase rosette and facilitates chromosome alignment and bi-orientation establishment
Source: Sci Rep. 2018 Mar 1;8:3888. doi: 10.1038/s41598-018-22164-5 (PMC5832872; doi:10.1038/s41598-018-22164-5)
Supplement: Supplementary file 1 — Supplementary Information [file 41598_2018_22164_MOESM1_ESM.pdf]

Supplementary Information

Lateral attachment of kinetochores to microtubules is enriched in prometaphase rosette and facilitates chromosome alignment and bi-orientation establishment

Go Itoh[1,2,*], Masanori Ikeda[1,*], Kenji Iemura[1], Mohammed Abdullahel Amin[1,†], Sei Kuriyama[2], Masamitsu Tanaka[2], Tokuko Haraguchi[3,4,5], Hiroko Osakada[3], Natsuki Mizuno[1], and Kozo Tanaka[1]

[1]Department of Molecular Oncology, Institute of Development, Aging and Cancer, Tohoku University, Sendai 980-8575, Japan.
[2]Department of Molecular Medicine and Biochemistry, Akita University Graduate School of Medicine, Akita 010-8543, Japan.
[3]Advanced ICT Research Institute, National Institute of Information and Communications Technology (NICT), Kobe 651-2492, Japan.
[4]Graduate School of Frontier Biosciences, Osaka University, Suita 565-0871, Japan.
[5]Graduate School of Science, Osaka University, Toyonaka 560-0043, Japan.

[*]These authors contributed equally to this work.
[†] Current address: Department of Cell and Molecular Biology, Feinberg School of Medicine, Northwestern University, Chicago, IL 60611, USA.
Correspondence and requests for materials should be addressed to K.T. (email: kozo.tanaka.d2@tohoku.ac.jp)

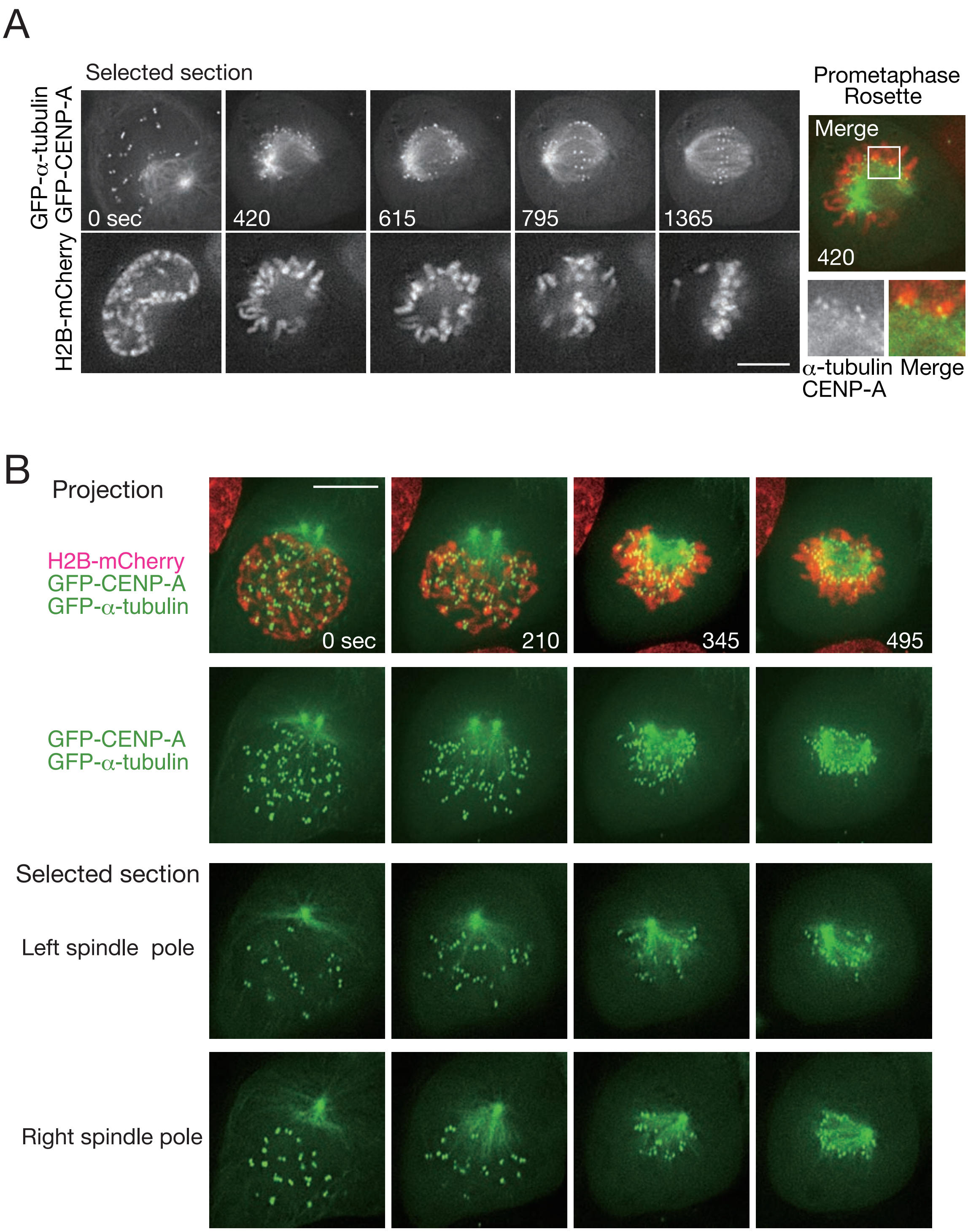

Supplementary Figure S1. Formation of the prometaphase rosette. (A) HeLa cells expressing EGFP–α-tubulin (green), EGFP–CENP-A (green), and H2B–mCherry (red) were imaged at 15 s intervals, starting from NEBD. A selected section is shown as left panels. The right panel shows the merged image at 420 sec. Magnified view of the prometaphase rosette boxed in the panel is shown in insets. Scale bar: 10 μm. (B) HeLa cells expressing EGFP–α-tubulin (green), EGFP–CENP-A (green), and H2B–mCherry (red) were imaged at 15 s intervals, starting from NEBD. Projected images are shown in the upper panels, while selected sections containing respective spindle poles are shown in the lower panels. Scale bar: 10 μm.

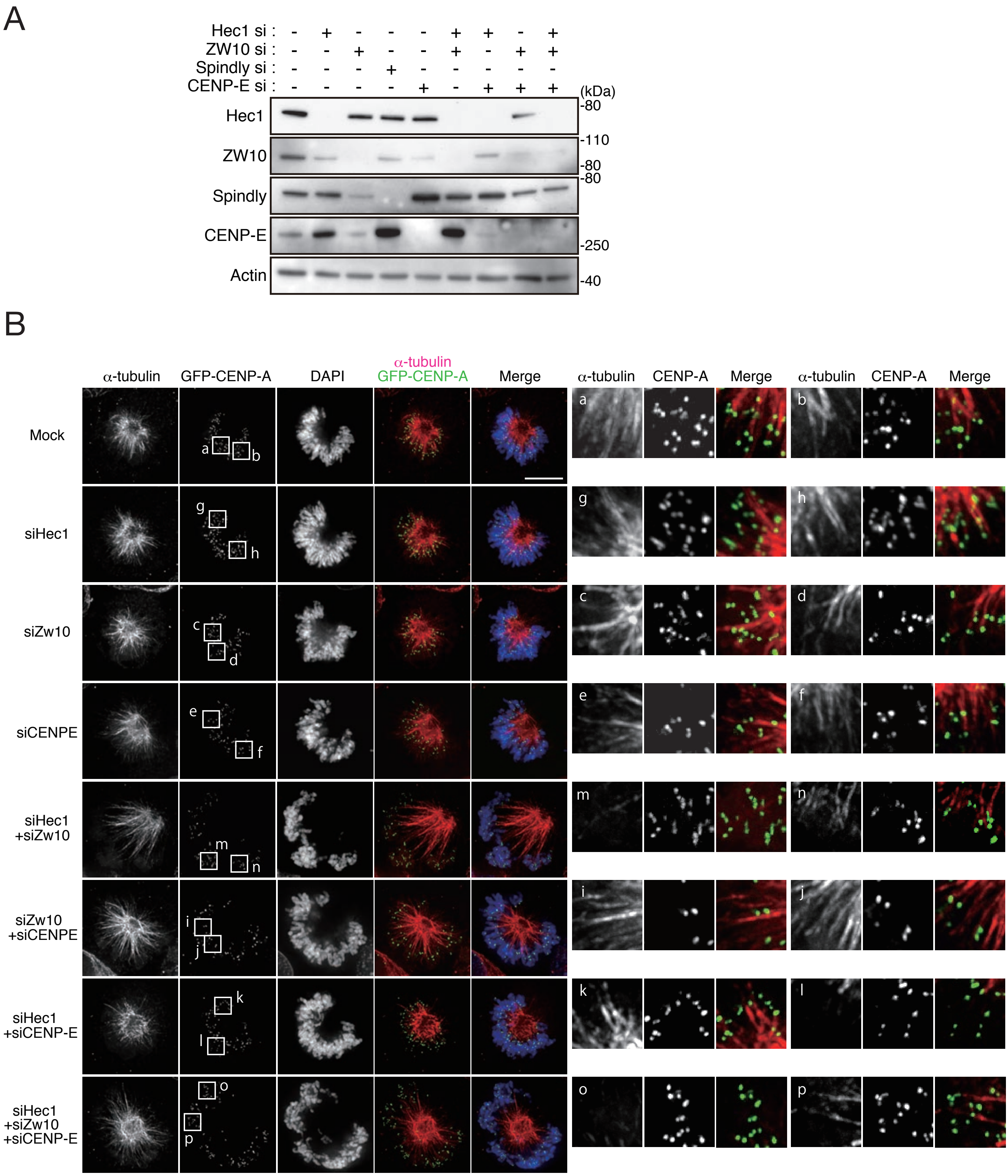

Supplementary Figure S2. Kinetochore-microtubule attachment in cells depleted of molecules related to lateral and end-on attachment during the rosette formation. (A) Efficiency of RNAi in HeLa cells. Lysate of cells transfected with an siRNA against each molecule indicated was subjected to immunoblot analysis using antibodies against each molecule as indicated. (B) Kinetochore-microtubule attachment in cells depleted of molecules related to lateral and end-on attachment during the rosette

formation. HeLa cells expressing EGFP–CENP-A (green) were immunostained with an antibody against α-tubulin (red). DNA was stained with DAPI (blue). Magnified view of kinetochore pairs boxed in the panel is shown in insets. Scale bar: 10 μm.

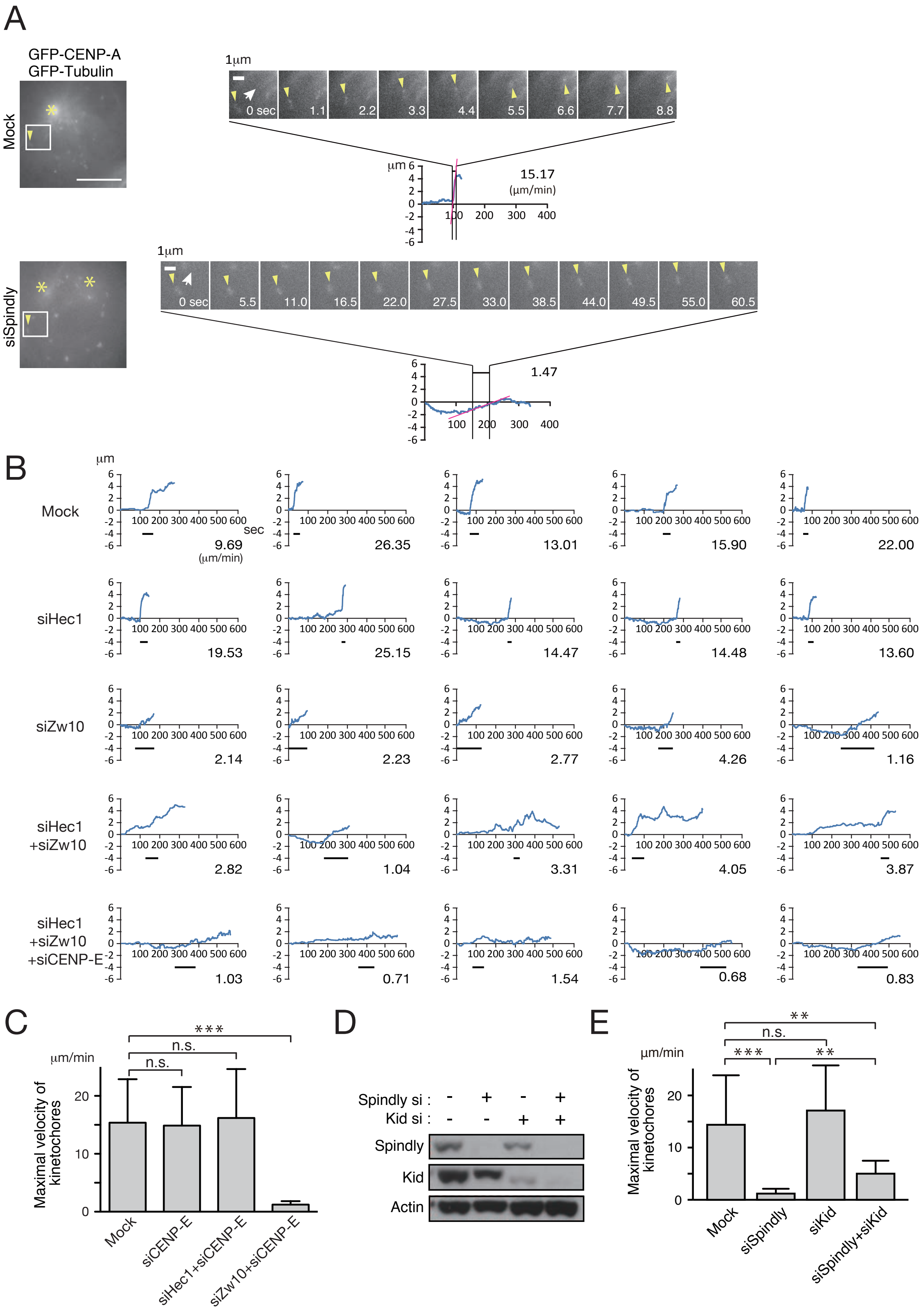
A
GFP-CENP-A
GFP-Tubulin
Mock
1μm
0 sec
1.1
2.2
3.3
4.4
5.5
6.6
7.7
8.8
μm
15.17
(μm/min)
100 200 300 400
siSpindly
1μm
0 sec
5.5
11.0
16.5
22.0
27.5
33.0
38.5
44.0
49.5
55.0
60.5
1.47
B
μm
sec
Mock
9.69
(μm/min)
26.35
13.01
15.90
22.00
siHec1
19.53
25.15
14.47
14.48
13.60
siZw10
2.14
2.23
2.77
4.26
1.16
siHec1
+siZw10
2.82
1.04
3.31
4.05
3.87
siHec1
+siZw10
+siCENP-E
1.03
0.71
1.54
0.68
0.83
100 200 300 400 500 600
C
μm/min
Maximal velocity of kinetochores
***
n.s.
n.s.
Mock
siCENP-E
siHec1+siCENP-E
siZw10+siCENP-E
D
Spindly si : - + - +
Kid si : - - + +
Spindly
Kid
Actin
E
μm/min
Maximal velocity of kinetochores
**
n.s.
***
**
Mock
siSpindly
siKid
siSpindly+siKid

F

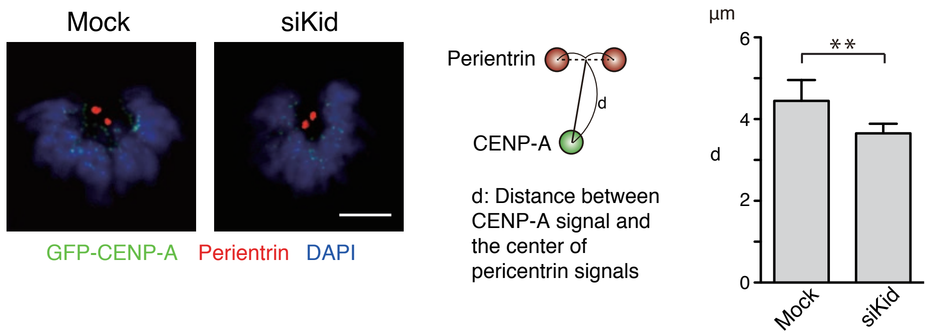

Supplementary Figure S3. Effect of the knockdown of molecules related to lateral and end-on attachment on the formation of the prometaphase rosette. (A) Kinetochore motion during rosette formation. HeLa cells expressing EGFP–CENP-A and EGFP–α-tubulin was imaged at 550 msec intervals after NEBD. Representative examples of kinetochore motion in a mock-treated cell and a Spindly-depleted cell are shown. Kinetochore positions are shown by yellow arrowheads. Kinetochore displacement from the starting position is shown in each graph, representing the motion towards the spindle pole (direction shown by an arrow; the position of the spindle pole is shown by an asterisk) as a positive value. Maximal kinetochore velocity (slope of the magenta line) was calculated by displacement during indicated frames. Scale bar: 10 µm. (B) Examples of kinetochore displacement soon after NEBD, measured as in (A), in cells depleted of Hec1, Zw10, or CENP-E is shown. Maximal velocity of kinetochore, calculated as the slope during the period indicated as a bar, is shown. At least 14 kinetochores from 3 cells were tracked for each condition. Error bars represent S.D. ***$P<0.0005$ (Student's t-test). n.s., not statistically significant. (C) Maximal velocity of kinetochores soon after NEBD in cells depleted of Hec1, Zw10, or CENP-E, measured as in (A). At least 13 kinetochores from 3 cells were tracked for each condition. Representative data from three independent experiments are shown. Error bars represent S.D. (D) Depletion of Spindly and/or Kid in HeLa cells. Lysate of cells transfected with an siRNA against each molecule indicated was subjected to immunoblot analysis using antibodies as indicated. (E) Maximal velocity of kinetochores soon after NEBD in cells depleted of Spindly and/or Kid, measured as in (A). At least 15 kinetochores from 3 cells were tracked for each condition. Representative data from three independent experiments are shown. Error bars represent S.D. **$P<0.005$; ***$P<0.0005$ (Student's t-test). n.s., not statistically significant. (F) Size of the prometaphase rosette in Kid-depleted cells. HeLa cells expressing EGFP-CENP-A (green) were immunostained with an antibody against pericentrin (red). DNA was stained with DAPI (blue). Representative images are shown in the left panels. Scale bar: 5 µm. The right graph shows distance between CANP-A signal and the center of pericentrin signals (d), as schematically shown. At least 23 kinetochores from 7 cells were observed for each condition. Representative data from three independent experiments are shown. Error bars represent S.D. **$P<0.005$ (Student's t-test).

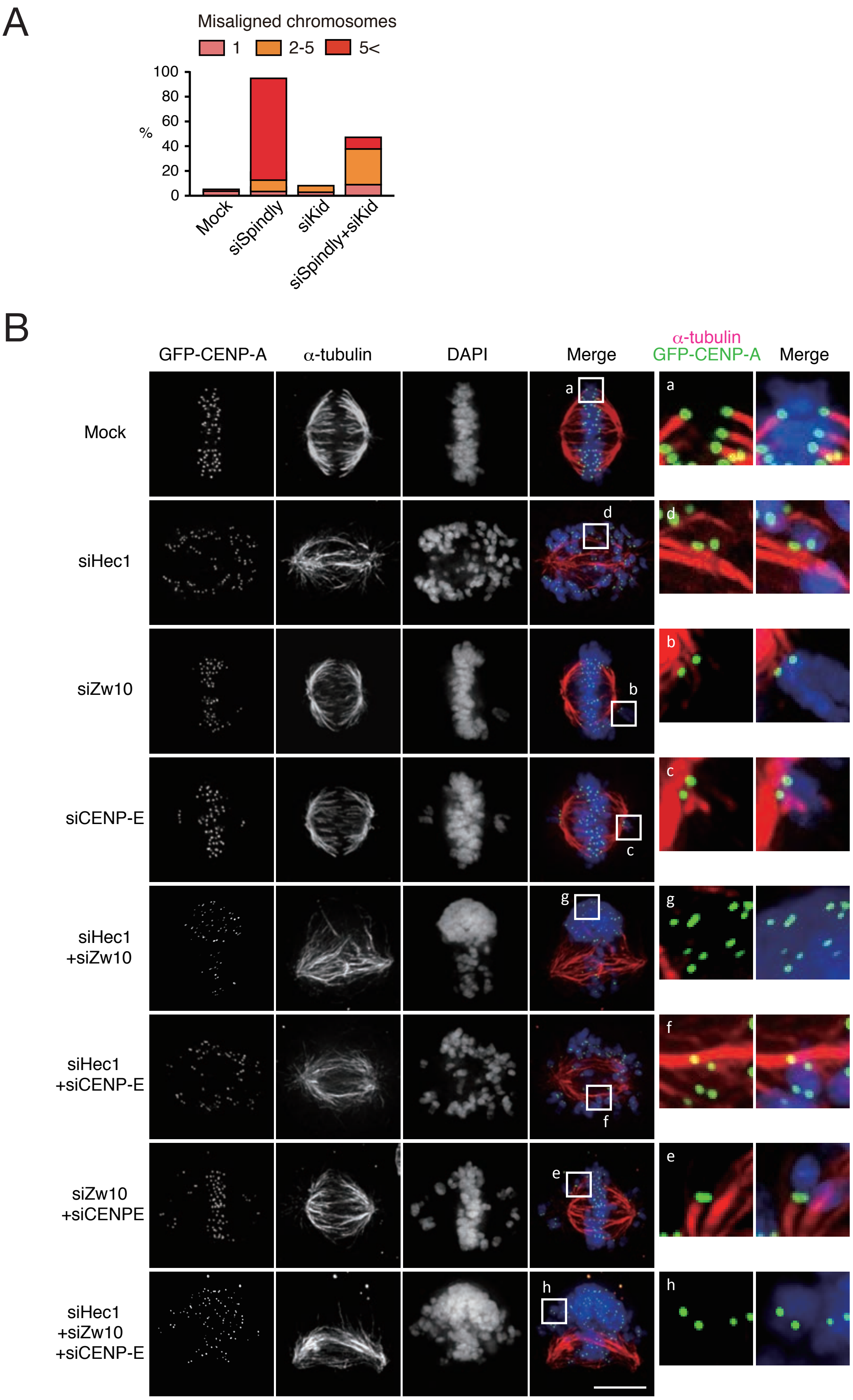

Supplementary Figure S4. Kinetochore-microtubule attachment in cells depleted of molecules related to lateral and end-on attachment in pseudo-metaphase. (A) Proportion of cells with misaligned chromosomes when they were depleted of Spindly and/or Kid. Cells were treated and counted as in Fig. 6B. At least100 cells were observed for each condition. Representative data from three independent experiments are shown. (B) HeLa cells expressing EGFP–CENP-A (green) were immunostained with an antibody against tubulin (red). DNA was stained with DAPI (blue). Magnified view of kinetochores boxed in the panel is shown in insets. Scale bar: 10 μm.

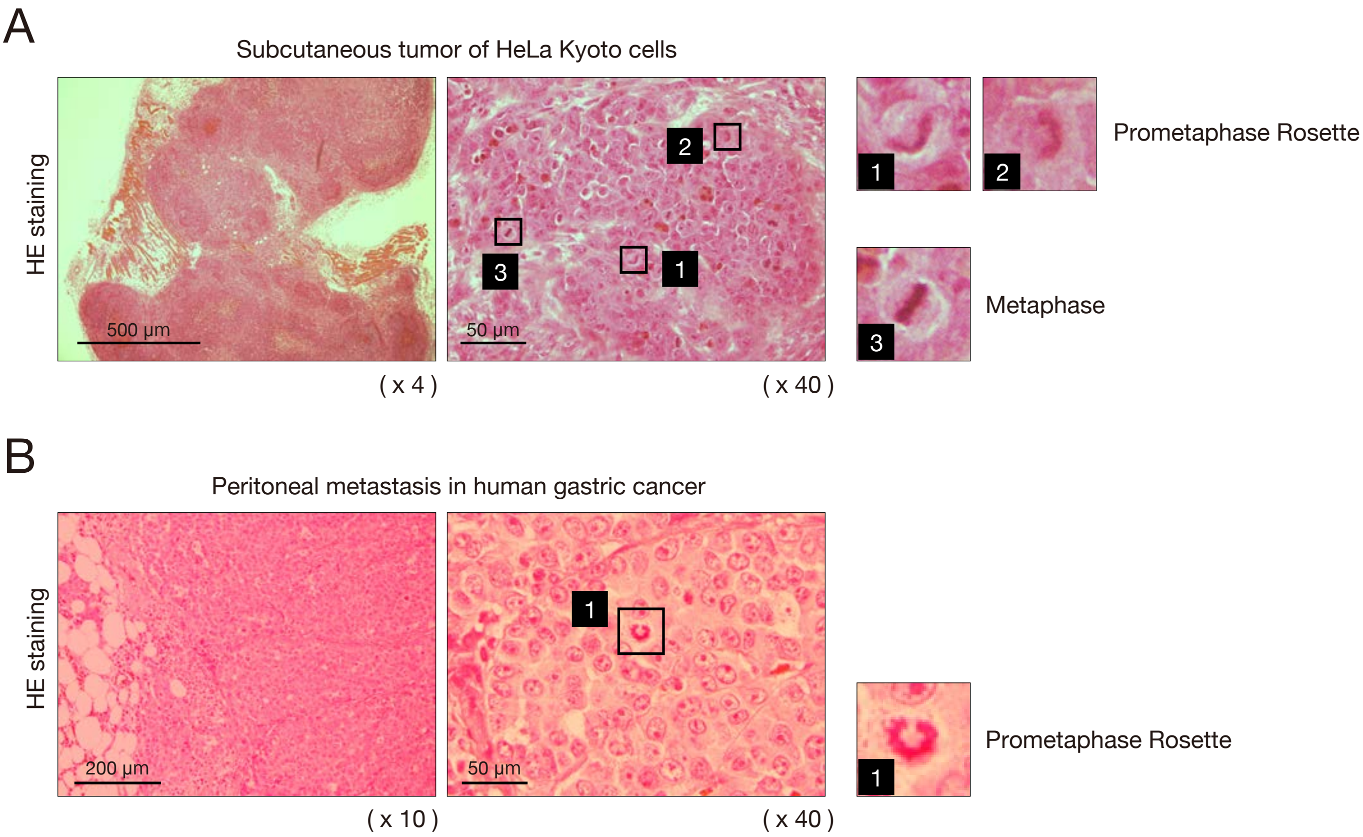

Supplementary Figure S5. Prometaphase rosettes in tissue samples of cancer cells. (A) HE staining of subcutaneous tumor of HeLa Kyoto cells. Insets show mitotic cells showing the prometaphase rosette (1, 2) or in metaphase (3). (B) HE staining of peritoneal metastasis in human gastric cancer. Inset shows a mitotic cell showing a prometaphase rosette (1).

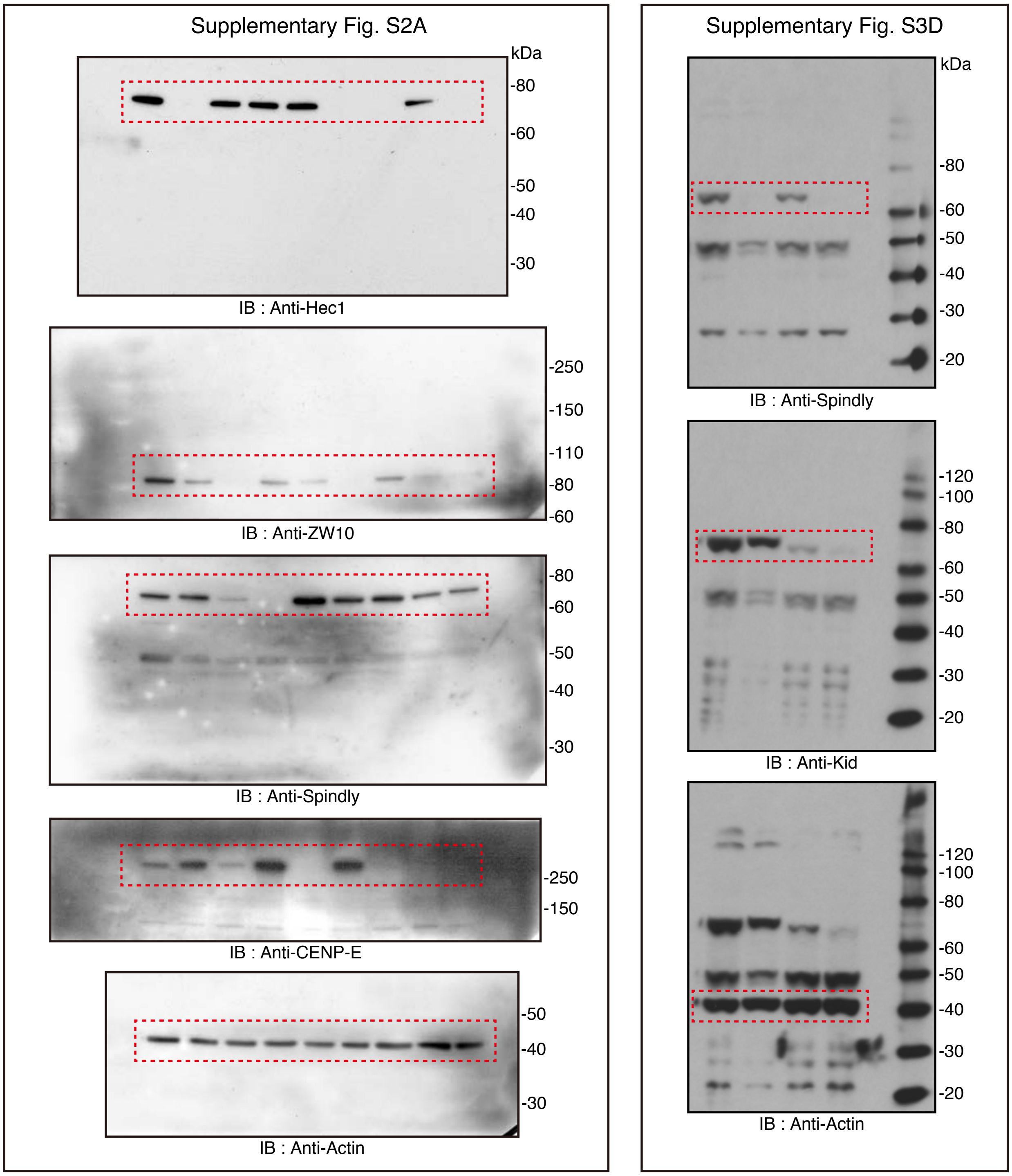

Supplementary Figure S6. Uncropped images of immunoblots.

Supplementary Movies

Supplementary Movie 1. Live imaging of a HeLa cells expressing EGFP–α-tubulin (green), EGFP–CENP-A (green), and H2B–mCherry (red) in which centrosomes reside at the same side at NEBD. Stills of this movie are displayed in Fig. 1A(i). Images were collected every 1 min, and the display rate is 10 frames/s.

Supplementary Movie 2. Live imaging of a HeLa cells expressing EGFP–α-tubulin (green), EGFP-CENP-A (green), and H2B-mCherry (red) in which centrosomes reside at opposite sides at NEBD. Stills of this movie are displayed in Fig. 1A(ii). Images were collected every 1 min, and the display rate is 10 frames/s.

Supplementary Movie 3. Live imaging of a mock-treated HeLa cell expressing EGFP–α-tubulin (green), EGFP–CENP-A (green), and H2B–mCherry (red) forming a prometaphase rosette. Stills of this movie are displayed in Fig. 5A. Images were collected every 15 sec, starting from NEBD, and the display rate is 10 frames/s.

Supplementary Movie 4. Live imaging of a Hec1-depleted HeLa cell expressing EGFP–α-tubulin (green), EGFP–CENP-A (green), and H2B–mCherry (red) forming a prometaphase rosette. Stills of this movie are displayed in Fig. 5A. Images were collected every 15 sec, starting from NEBD, and the display rate is 10 frames/s.

Supplementary Movie 5. Live imaging of a Zw10-depleted HeLa cell expressing EGFP–α-tubulin (green), EGFP–CENP-A (green), and H2B–mCherry (red) forming a prometaphase rosette. Stills of this movie are displayed in Fig. 5A. Images were collected every 15 sec, starting from NEBD, and the display rate is 10 frames/s.

Supplementary Movie 6. Live imaging of a Spindly-depleted HeLa cell expressing EGFP–α-tubulin (green), EGFP–CENP-A (green), and H2B–mCherry (red) forming the prometaphase rosette. Stills of this movie are displayed in Fig. 5A. Images were collected every 15 sec, starting from NEBD, and the display rate is 10 frames/s.

Supplementary Movie 7. Live imaging of a HeLa cell expressing EGFP–α-tubulin (green), EGFP–CENP-A (green), and H2B–mCherry (red) treated with ZM-447439 in early prometaphase. Stills of this movie are displayed in Fig. 5A. Images were collected every 15 sec, starting from NEBD, and the display rate is 10 frames/s.

Supplementary Movie 8. Live imaging of a HeLa cell expressing EGFP–α-tubulin (green), EGFP–CENP-A (green), and H2B–mCherry (red) depleted of Hec1 and Zw10 forming a prometaphase rosette. Stills of this movie are displayed in Fig. 5A. Images were collected every 15 sec, starting from NEBD, and the display rate is 10 frames/s.

Supplementary Movie 9. Live imaging of a HeLa cell expressing EGFP–α-tubulin (green), EGFP–CENP-A (green), and H2B–mCherry (red) depleted of Hec1, Zw10, and CENP-E in early prometaphase. Stills of this movie are displayed in Fig. 5A. Images were collected every 15 sec, starting from NEBD, and the display rate is 10 frames/s.

Supplementary Movie 10. Live imaging of a HeLa cell expressing EGFP–α-tubulin (green), EGFP–CENP-A (green), and H2B–mCherry (red) treated with taxol, forming a prometaphase rosette. Stills of this movie are displayed in Fig. 5A. Images were collected every 15 sec, starting from NEBD, and the display rate is 10 frames/s.

Supplementary Movie 11. Live imaging of a Zw10-depleted HeLa cell expressing EGFP–α-tubulin (green), EGFP–CENP-A (green), and H2B–mCherry (red) treated with taxol in early prometaphase. Stills of this movie are displayed in Fig. 5A. Images were collected every 15 sec, starting from NEBD, and the display rate is 10 frames/s.

Supplementary Movie 12. Live imaging of a mock-treated HeLa cell expressing EGFP–α-tubulin (green), EGFP–CENP-A (green), and H2B–mCherry (red) during mitosis. Stills of this movie are displayed in Fig. 6A. Images were collected every 1 min, starting from NEBD, and the display rate is 10 frames/s.

Supplementary Movie 13. Live imaging of a Hec1-depleted HeLa cell expressing EGFP–α-tubulin (green), EGFP–CENP-A (green), and H2B–mCherry (red) during

mitosis. Stills of this movie are displayed in Fig. 6A. Images were collected every 1 min, starting from NEBD, and the display rate is 10 frames/s.

Supplementary Movie 14. Live imaging of a Zw10-depleted HeLa cell expressing EGFP–α-tubulin (green), EGFP–CENP-A (green), and H2B–mCherry (red) during mitosis. Stills of this movie are displayed in Fig. 6A. Images were collected every 1 min, starting from NEBD, and the display rate is 10 frames/s.

Supplementary Movie 15. Live imaging of a Spindly-depleted HeLa cell expressing EGFP–α-tubulin (green), EGFP–CENP-A (green), and H2B–mCherry (red) during mitosis. Stills of this movie are displayed in Fig. 6A. Images were collected every 1 min, starting from NEBD, and the display rate is 10 frames/s.

Supplementary Movie 16. Live imaging of a CENP-E-depleted HeLa cell expressing EGFP–α-tubulin (green), EGFP–CENP-A (green), and H2B–mCherry (red) during mitosis. Stills of this movie are displayed in Fig. 6A. Images were collected every 1 min, starting from NEBD, and the display rate is 10 frames/s.

Supplementary Movie 17. Live imaging of a Zw10, Hec1-depleted HeLa cell expressing EGFP–α-tubulin (green), EGFP–CENP-A (green), and H2B–mCherry (red) during mitosis. Stills of this movie are displayed in Fig. 6A. Images were collected every 1 min, starting from NEBD, and the display rate is 10 frames/s.

Supplementary Movie 18. Live imaging of a Spindly, Hec1-depleted HeLa cell expressing EGFP–α-tubulin (green), EGFP–CENP-A (green), and H2B–mCherry (red) during mitosis. Stills of this movie are displayed in Fig. 6A. Images were collected every 1 min, starting from NEBD, and the display rate is 10 frames/s.

Supplementary Movie 19. Live imaging of a CENP-E, Hec1-depleted HeLa cell expressing E–GFP-α-tubulin (green), EGFP–CENP-A (green), and H2B–mCherry (red) during mitosis. Stills of this movie are displayed in Fig. 6A. Images were collected every 1 min, starting from NEBD, and the display rate is 10 frames/s.

Supplementary Movie 20. Live imaging of a Zw10, CENP-E, Hec1-depleted HeLa cell expressing EGFP–α-tubulin (green), EGFP–CENP-A (green), and H2B–mCherry (red) during mitosis. Stills of this movie are displayed in Fig. 6A. Images were collected every 1 min, starting from NEBD, and the display rate is 10 frames/s.

Supplementary Movie 21. Live imaging of a HeLa cell expressing EGFP–α-tubulin (green), EGFP–CENP-A (green), and H2B–mCherry (red) treated with ZM-447439 during mitosis. Stills of this movie are displayed in Fig. 6A. Images were collected every 1 min, starting from NEBD, and the display rate is 10 frames/s.

Supplementary Movie 22. Live imaging of a Hec1-depleted HeLa cell expressing EGFP–α-tubulin (green), EGFP–CENP-A (green), and H2B–mCherry (red) treated with ZM-447439 during mitosis. Stills of this movie are displayed in Fig. 6A. Images were collected every 1 min, starting from NEBD, and the display rate is 10 frames/s.
